# Supplementary material for: Nanoscale covalent organic frameworks for enhanced photocatalytic hydrogen production
Source: Nat Commun. 2024 Aug 1;15:6482. doi: 10.1038/s41467-024-50839-3 (PMC11294449; doi:10.1038/s41467-024-50839-3)
Supplement: Supplementary file 1 — Supplementary Information [file 41467_2024_50839_MOESM1_ESM.pdf]

Supporting Information

Nanoscale Covalent Organic Frameworks for Enhanced Photocatalytic Hydrogen Production

Wei Zhao[1], Liang Luo[1], Muyu Cong[2], Xueyan Liu[2], Zhiyun Zhang[2], Mounib Bahri[3], Boyu Li[1], Jing Yang[1], Miaojie Yu[1,2], Lunjie Liu[4], Yu Xia[4], Nigel D Browning[3], Wei-Hong Zhu[2], Weiwei Zhang*[2] and Andrew I. Cooper*[1]

[1] Leverhulme Research Centre for Functional Materials Design, Materials Innovation Factory and Department of Chemistry, University of Liverpool, Liverpool, UK.

[2] Key Laboratory for Advanced Materials and Institute of Fine Chemicals, School of Chemistry and Molecular Engineering, East China University of Science and Technology, Shanghai, China

[3] Albert Crewe Centre for Electron Microscopy, University of Liverpool, Liverpool, L69 3GL, UK.

[4] Department of Materials Science and Engineering, Southern University of Science and Technology, Shenzhen, 518055, China.

Table of contents

1. Characterization of COFs 2

2. References 72

1. Characterization of COFs

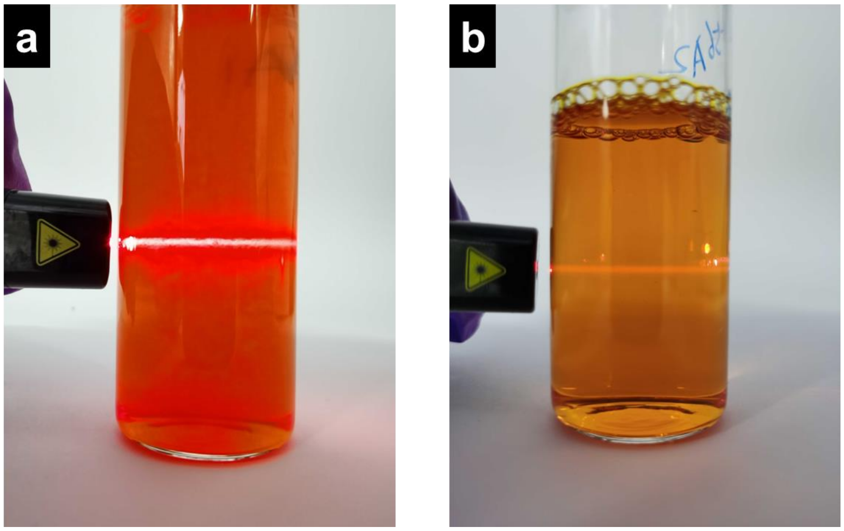

Supplementary Figure 1. Digital images of nano-COFs showing Willis-Tyndall scattering behavior using a laser pointer. (a) TFP-BpyD nano-COF. (b) TFP-BD nano-COF.

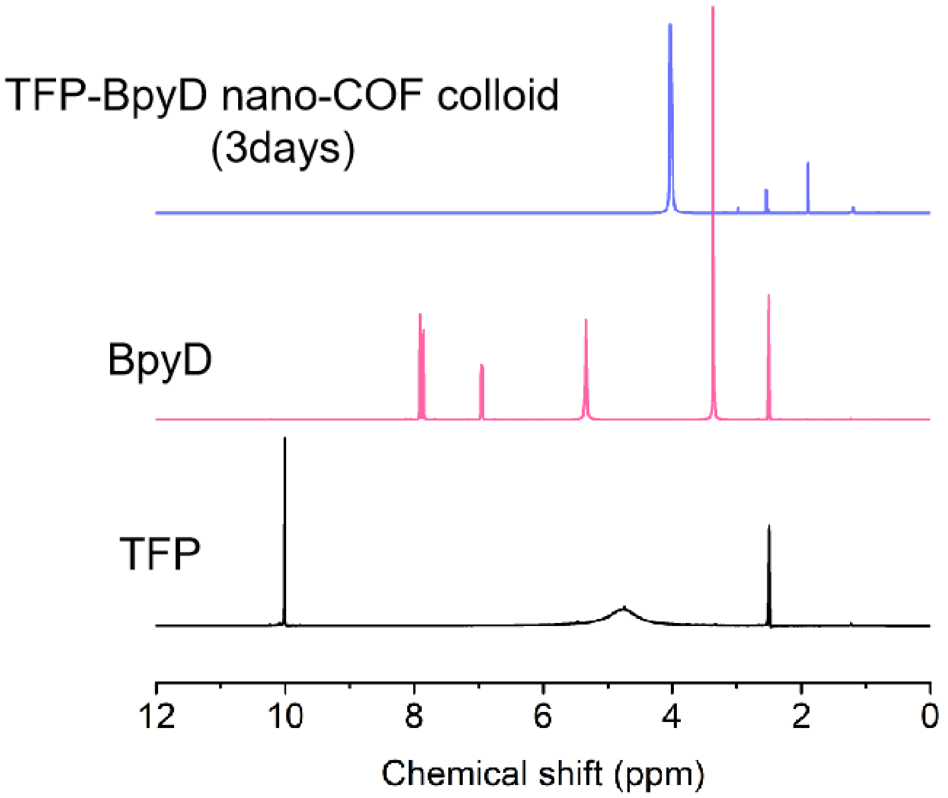

Supplementary Figure 2. ^{1}H NMR spectra of TFP-BpyD nano-COF colloid after 3-days reaction time, as compared with TFP and BpyD monomers. The peaks of colloid below 4 ppm are from the surfactants.

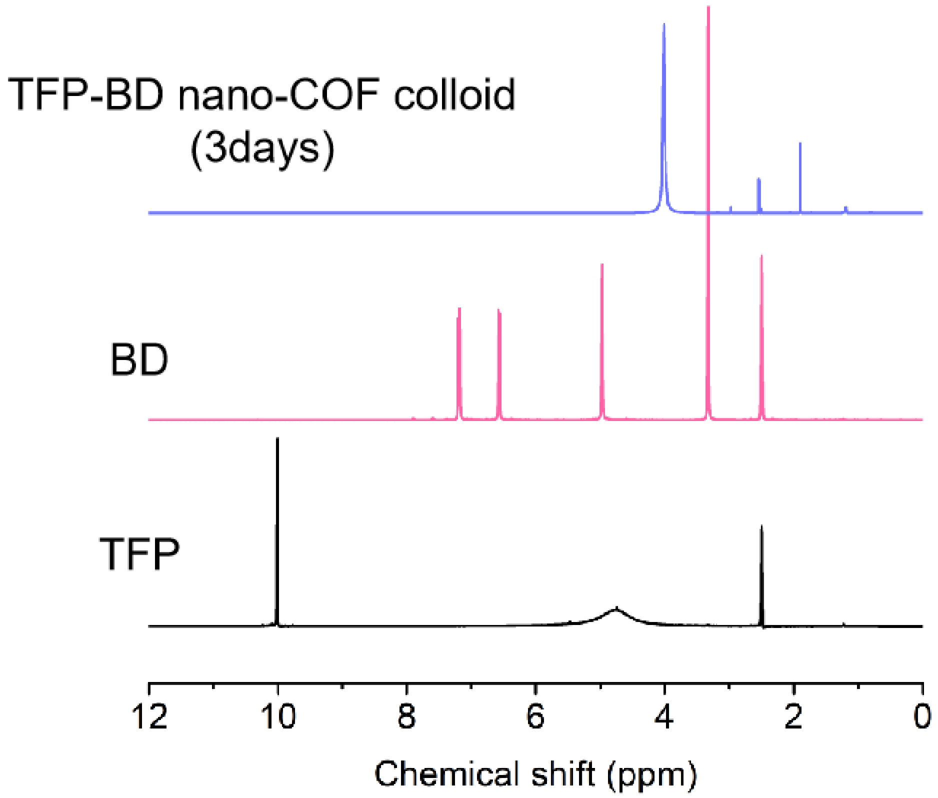

Supplementary Figure 3. ^{1}H NMR spectra of TFP-BD nano-COF colloid after 3-days reaction, as compared with TFP and BD monomers. The peaks of colloid below 4 ppm are from the surfactants.

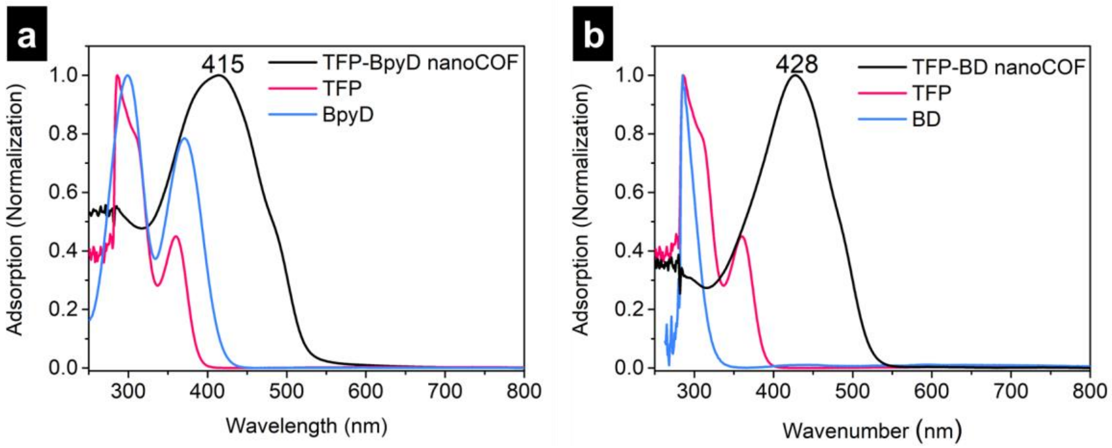

Supplementary Figure 4. UV-vis spectra of nano-COFs and corresponding starting materials. (a) TFP-BpyD nano-COF and (b) TFP-BD nano-COF showed different characteristic peaks. Conditions: 0.10 mL TFP-BpyD nano-COF, TFP-BD nano-COF, TFP, BpyD and BD solutions were diluted by water to a total volume of 3 mL.

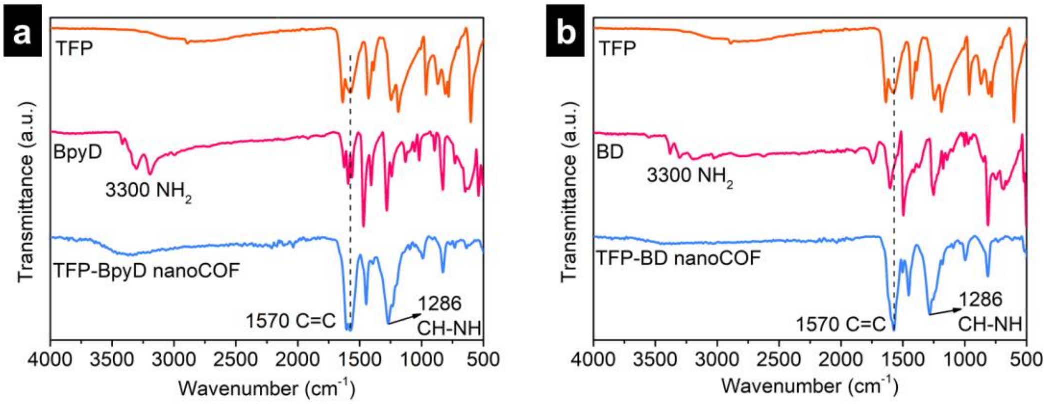

Supplementary Figure 5. FT-IR spectra of isolated nano-COFs and corresponding starting materials. (a) TFP-BpyD nano-COF. (b) TFP-BD nano-COF.

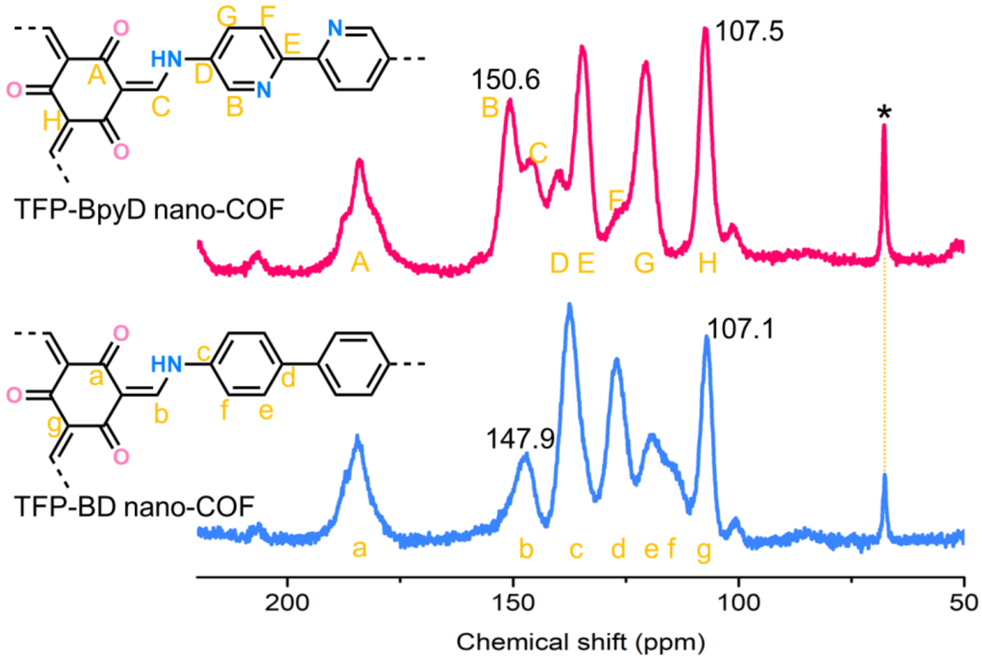

Supplementary Figure 6. ^{13}C CP-MAS solid-state NMR spectra of TFP-BpyD and TFP-BD nano-COF. The absence of the peak at 192 ppm in the ^{13}C CP-MAS NMR spectra indicated the total consumption of the starting materials. Both nano-COFs showed a clear signal near 184 and 148 ppm, corresponding to the carbonyl carbons and -**C**H-NH-, respectively. Spinning sidebands are denoted with asterisks.

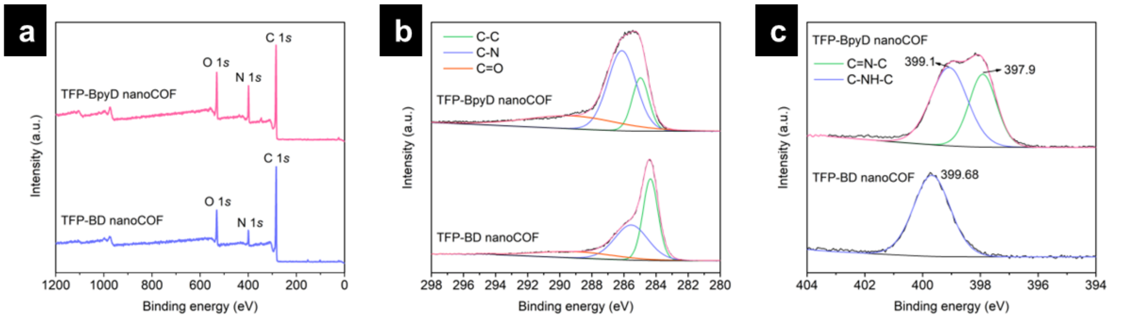

Supplementary Figure 7. XPS spectra of isolated nano-COFs. (a) Survey spectra of TFP-BpyD and TFP-BD nano-COF. High-resolution XPS spectra of (b) C 1*s* and (c) N 1*s* for TFP-BpyD and TFP-BD nano-COF. The full survey spectra confirmed the presence of C, N, and O elements of both nano-COFs. High-resolution XPS spectra of C 1*s* for both nano-COFs show the presence of ketoenamine C=O. High-resolution N 1*s* XPS spectrum of TFP-BpyD nano-COF can be deconvoluted into two individual peaks at 399.1 and 397.9 eV, attributable to the keto-enamine N (−C−HN−C−) and pyridinic N (−C=N−C−), respectively.

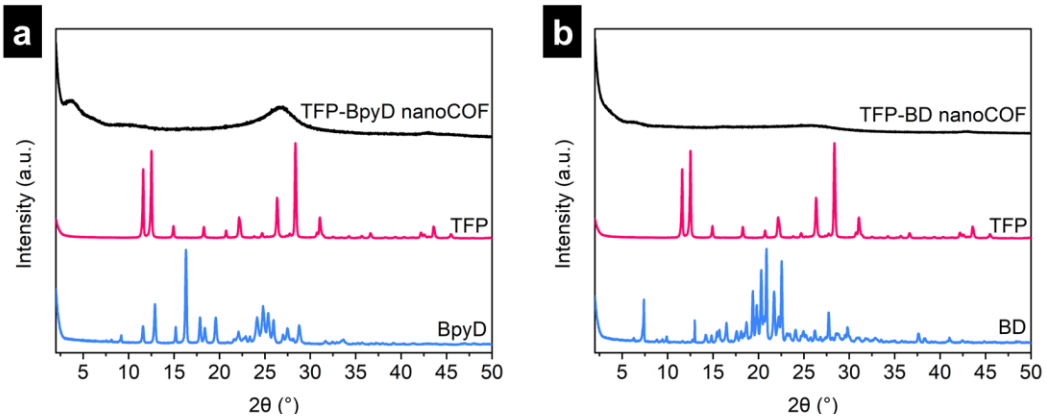

Supplementary Figure 8. PXRD patterns of isolated nano-COFs and corresponding starting materials. (a) TFP-BpyD nano-COF. (b)TFP-BD nano-COF.

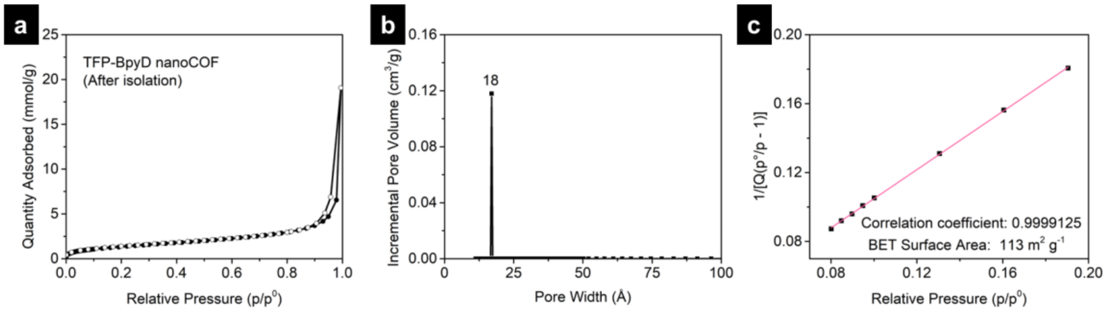

Supplementary Figure 9. N_2 sorption measurements of isolated TFP-BpyD nano-COF. (a) N_2 adsorption and desorption profiles at 77.3 K, (b) pore size distribution profile calculated by DFT and (c) BET surface area plot derived from N_2 sorption isotherm.

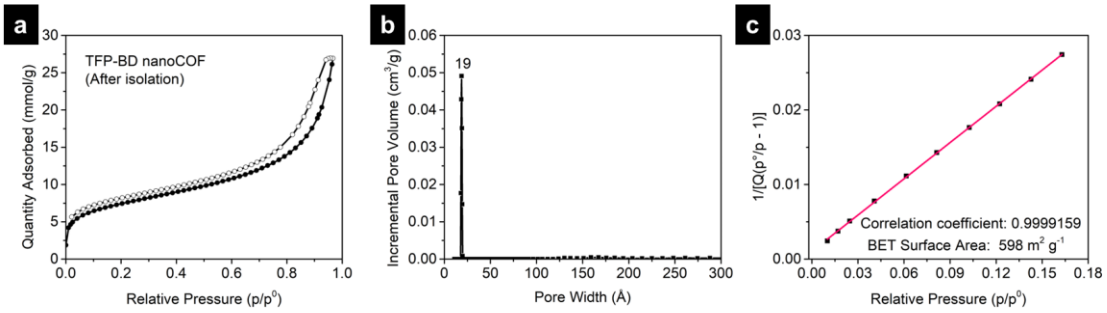

Supplementary Figure 10. N_2 sorption measurements of isolated TFP-BD nano-COF. (a) N_2 adsorption and desorption profiles at 77.3 K, (b) pore size distribution profile calculated by DFT and (c) BET surface area plot derived from N_2 sorption isotherm.

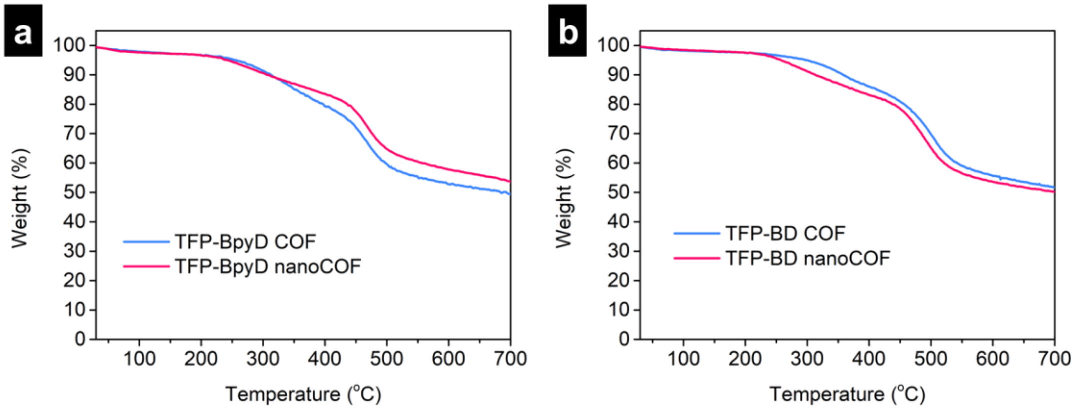

Supplementary Figure 11. TGA of COFs measured under N_2. (a) TFP-BpyD COF and TFP-BpyD nano-COF. (b) TFP-BD COF and TFP-BD nano-COF.

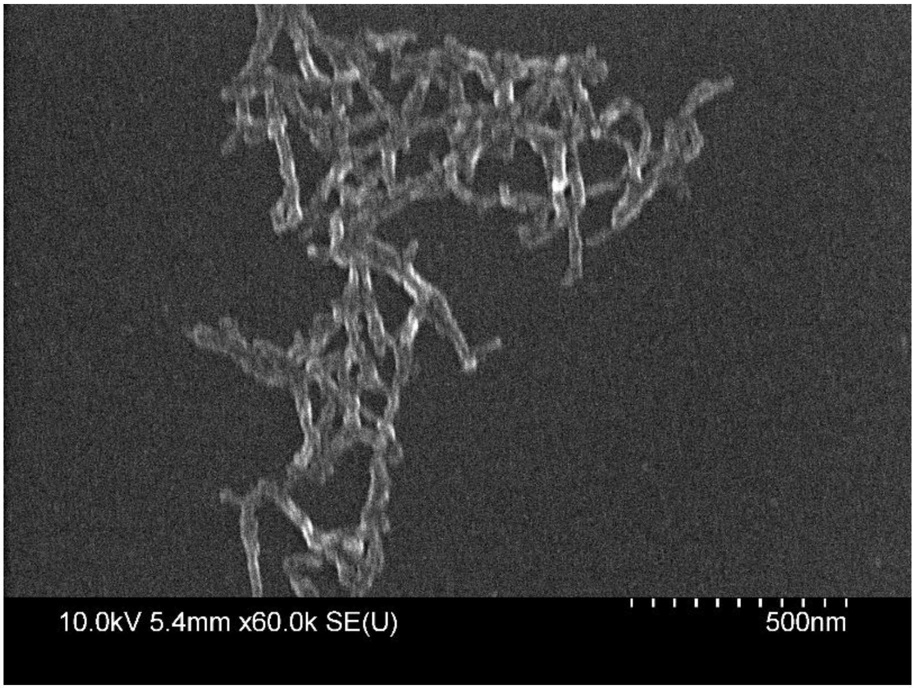

Supplementary Figure 12. SEM image of TFP-BpyD nano-COF.

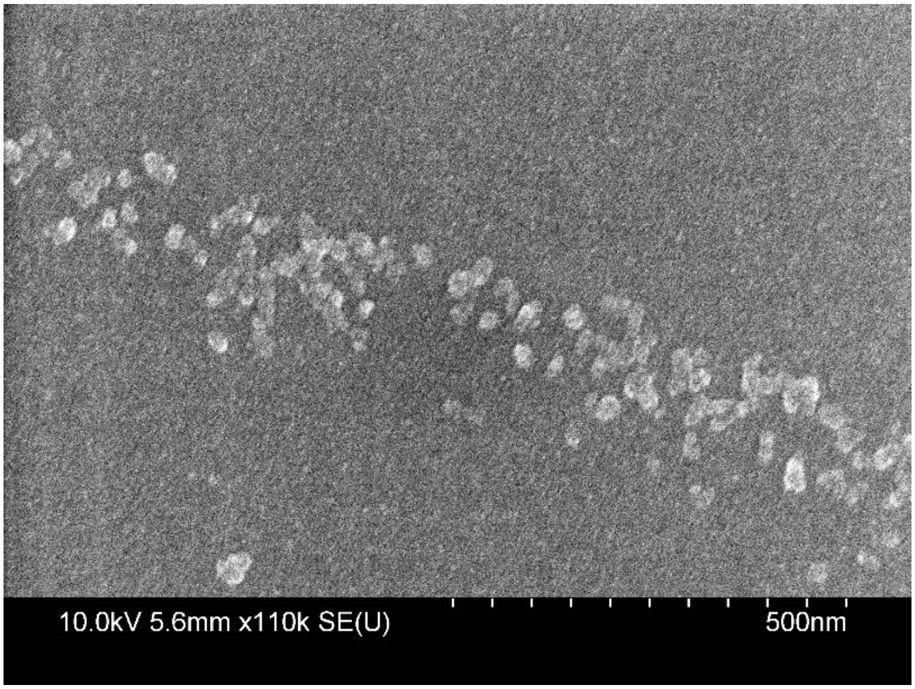

Supplementary Figure 13. SEM image of TFP-BD nano-COF.

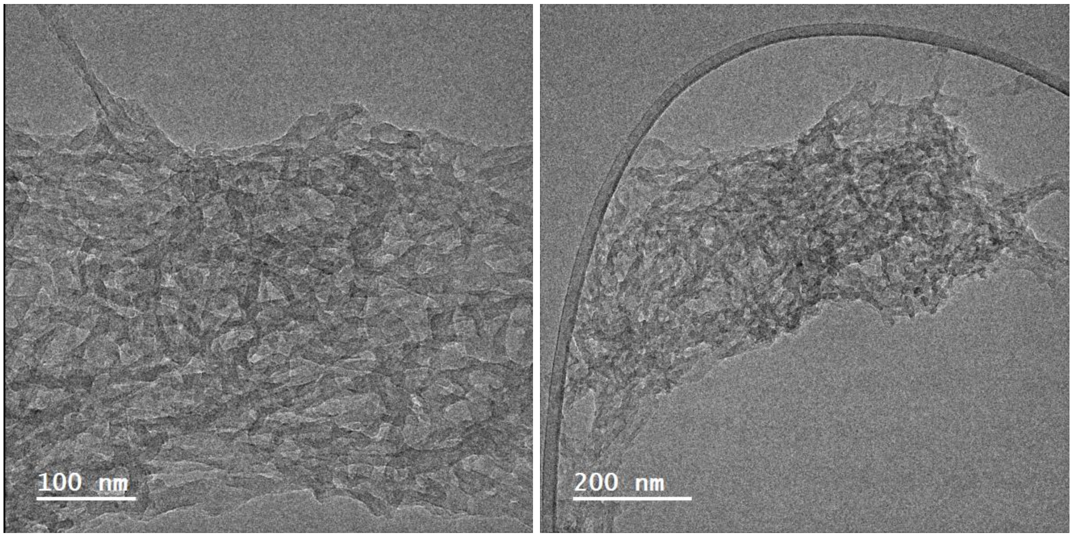

Supplementary Figure 14. TEM images of TFP-BpyD nano-COF.

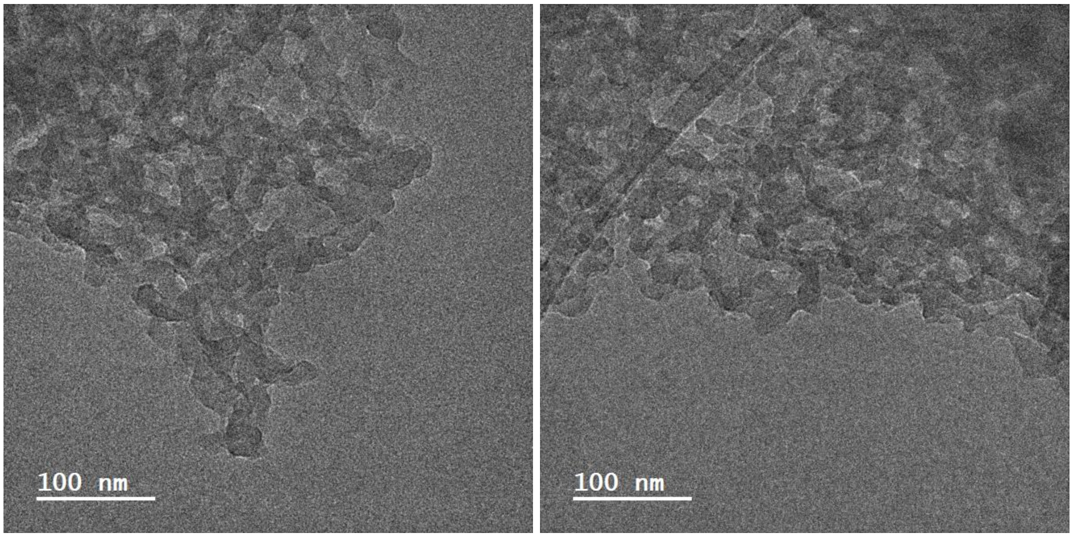

Supplementary Figure 15. TEM images of TFP-BD nano-COF.

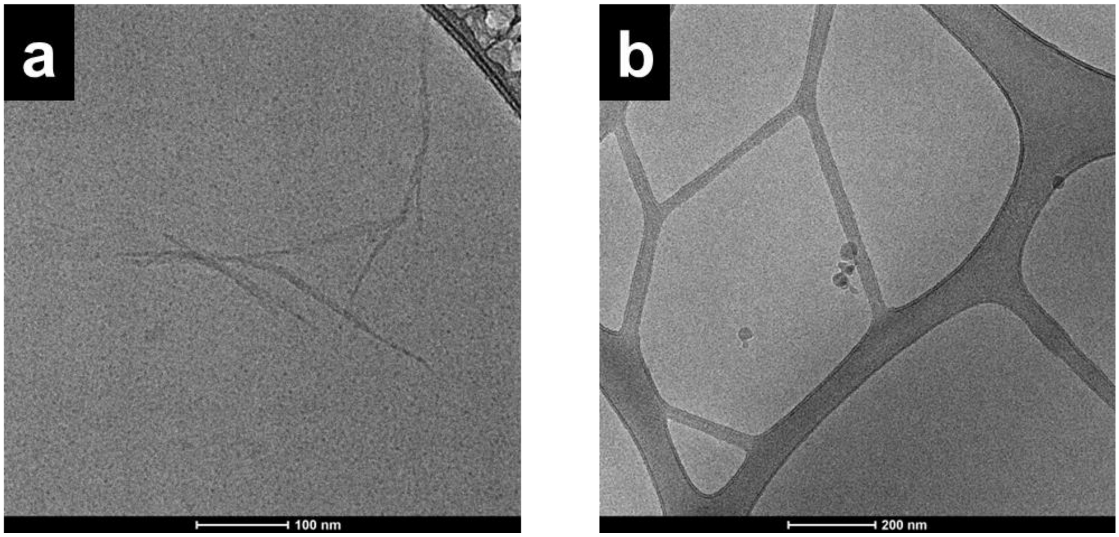

Supplementary Figure 16. Cryo-TEM images of nano-COFs. (a) TFP-BpyD nano-COF. (b) TFP-BD nano-COF.

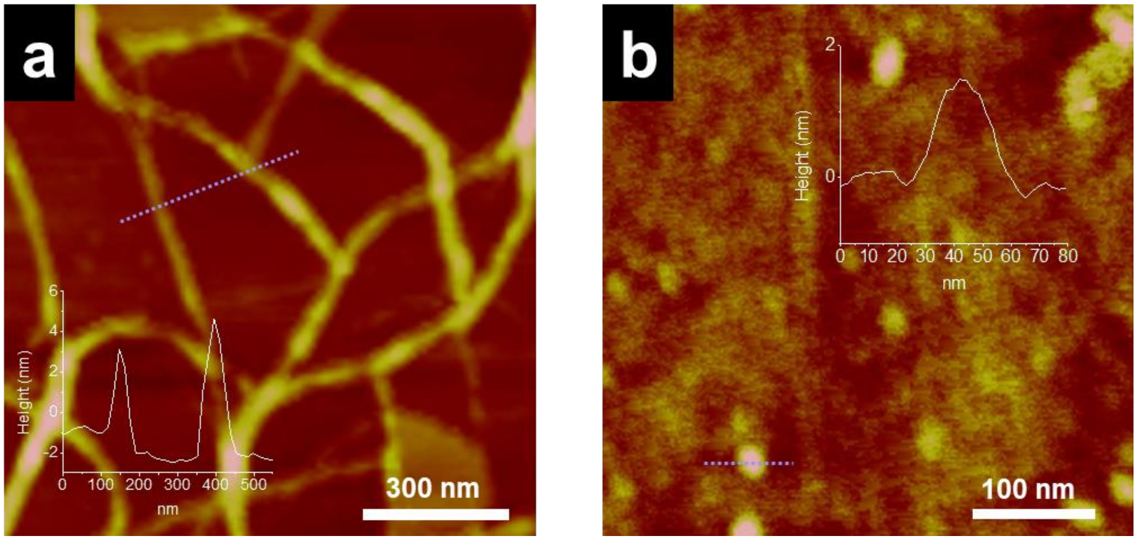

Supplementary Figure 17. AFM images of nano-COFs. (a) TFP-BpyD nano-COF. (b) TFP-BD nano-COF. Inserts: AFM height images with the corresponding cross-section analysis along the indicated dashed blue lines.

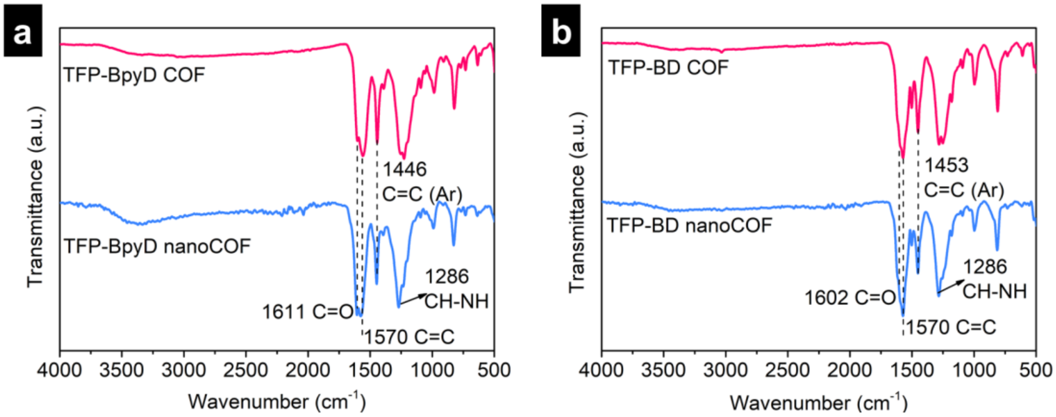

Supplementary Figure 18. FT-IR spectra of isolated nano-COFs compared with the analogous bulk COFs. (a) TFP-BpyD nano-COF and TFP-BpyD COF. (b) TFP-BD nano-COF and TFP-BD COF. FT-IR spectra revealed appearance of stretching bands at around 1570 cm^{-1} and 1286 cm^{-1}, which are assigned to the C=C and CH–NH bonds, indicating the formation of β-ketoenamine linkage in the two nano-COFs. However, because of the peak broadening in the extended structure, all C=O peaks of these COFs at around 1610 cm^{-1} were merged with the C=C stretching band at 1570 cm^{-1} and appeared as a shoulder.

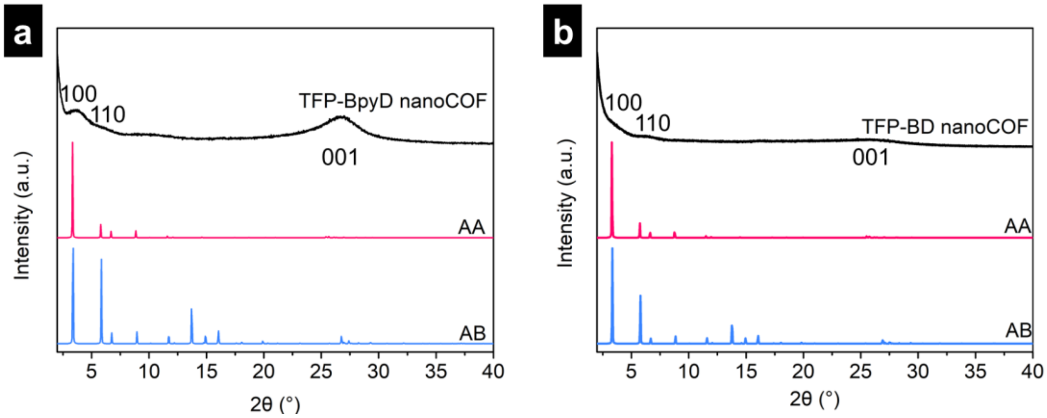

Supplementary Figure 19. PXRD patterns of isolated nano-COFs compared with the calculated models. (a) TFP-BpyD nano-COF. (b) TFP-BD nano-COF. Both nano-COFs show very little long-range order. The experimental PXRD pattern of TFP-BpyD nano-COF exhibited a relatively strong diffraction peak around at 3.4 °, which corresponds to the (100) reflection. Other reflection assignments are at best tentative given the very limited crystallinity in these nano-COFs.

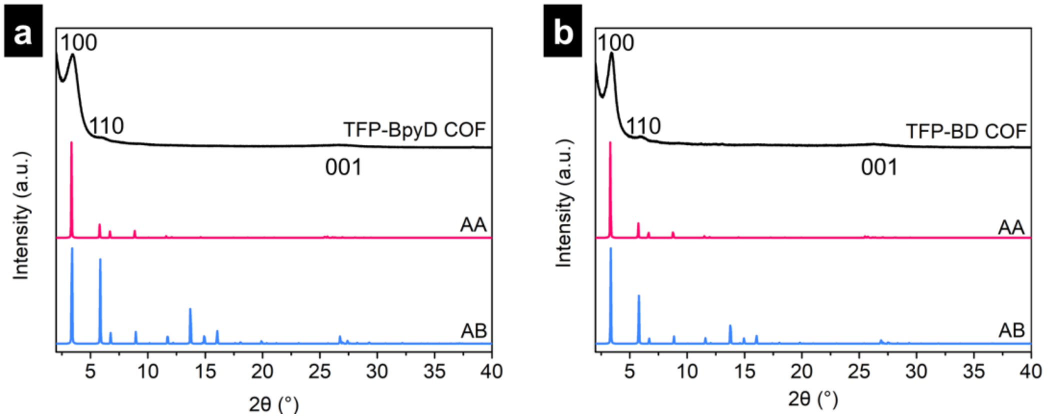

Supplementary Figure 20. Comparison of observed PXRD patterns of bulk COFs with the calculated models. (a) TFP-BpyD COF. (b) TFP-BD COF. These materials are significantly more crystalline than the nano-COF equivalents (Supplementary Figure 19). The experimental PXRD patterns of TFP-BpyD and TFP-BD COF both exhibited strong diffraction peaks around at 3.4 ° and relatively weak signals at 5.9 °, which correspond to the (100) and (110) reflections, respectively. The assignments for the weak reflections are at best tentative given the very limited crystallinity in these nano-COFs.

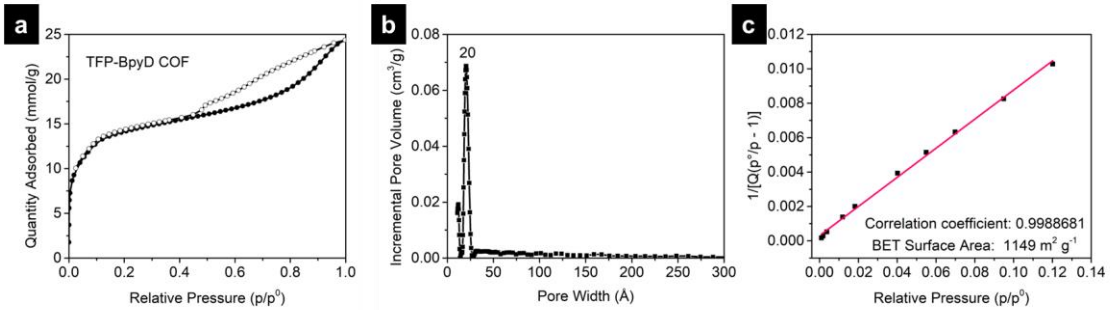

Supplementary Figure 21. N_2 sorption measurements of bulk TFP-BpyD COF. (a) N_2 adsorption and desorption profiles at 77.3 K, (b) pore size distribution profile calculated by DFT, and (c) BET surface area plot derived from N_2 sorption isotherm.

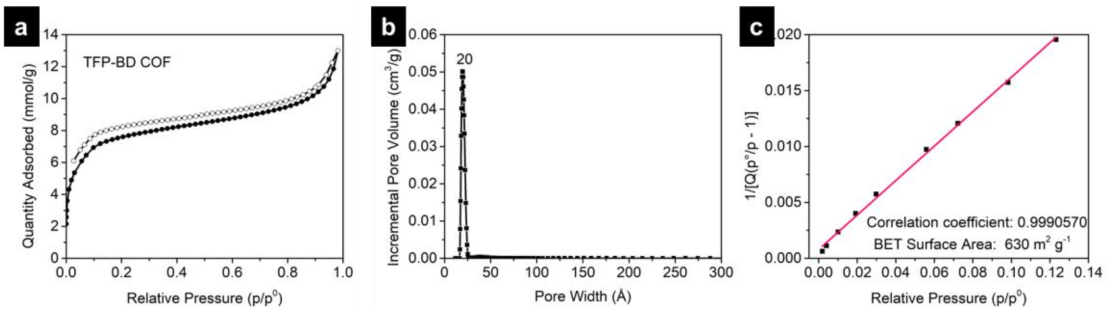

Supplementary Figure 22. N_2 sorption measurements of bulk TFP-BD COF. (a) N_2 adsorption and desorption profiles at 77.3 K, (b) pore size distribution profile calculated by DFT, and (c) BET surface area plot derived from N_2 sorption isotherm.

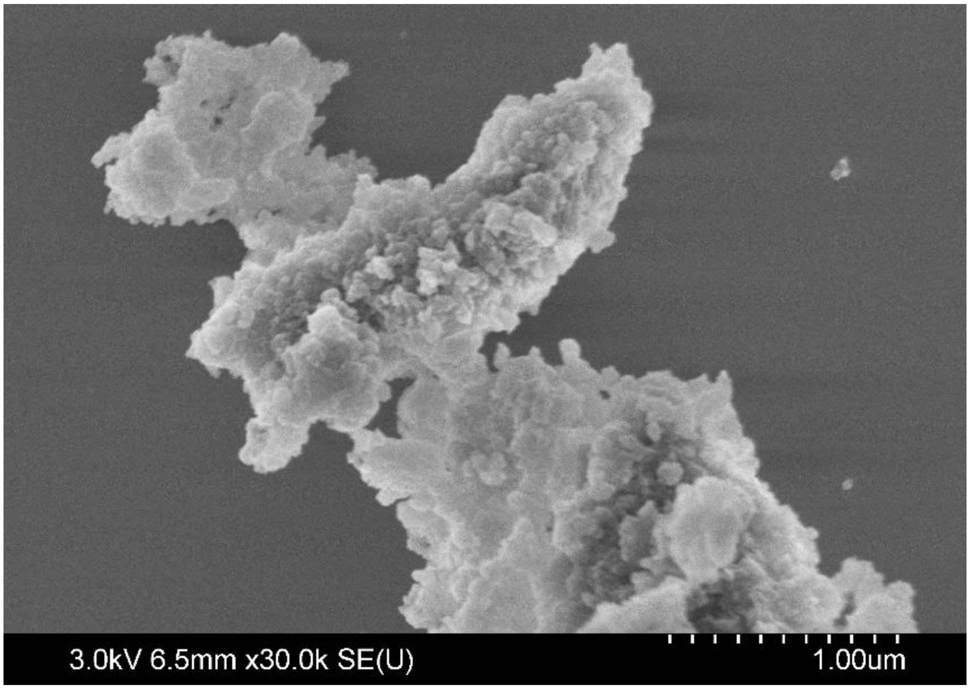

Supplementary Figure 23. SEM image of bulk TFP-BpyD COF.

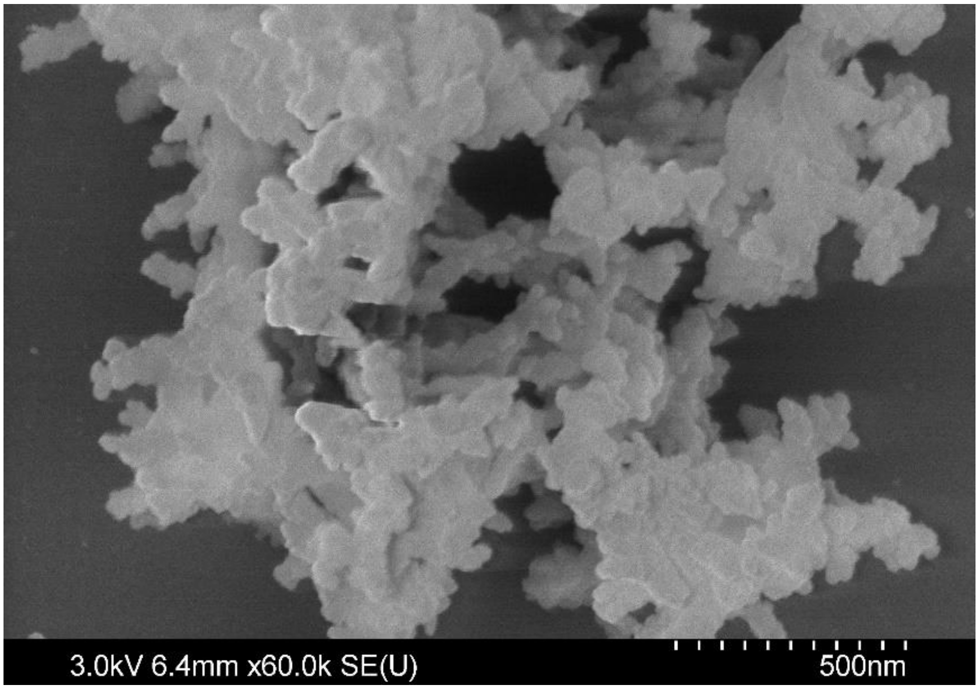

Supplementary Figure 24. **SEM image of bulk TFP-BD COF.**

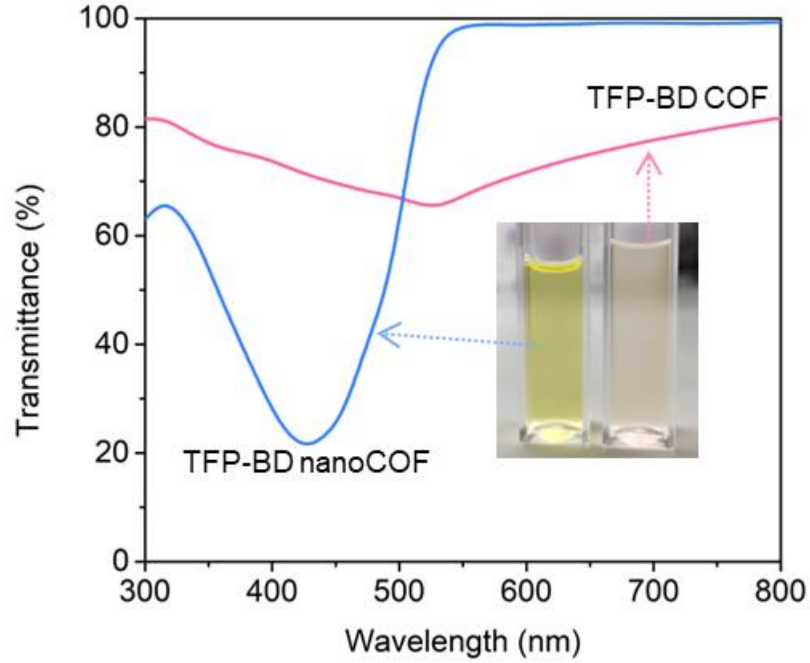

Supplementary Figure 25. Transmittance spectra of TFP-BD nano-COF as compared with bulk TFP-BD COF at the same concentration (5.67 µg / mL). TFP-BD nano-COF is more effective at light harvesting at same concentration than bulk TFP-BD COF.

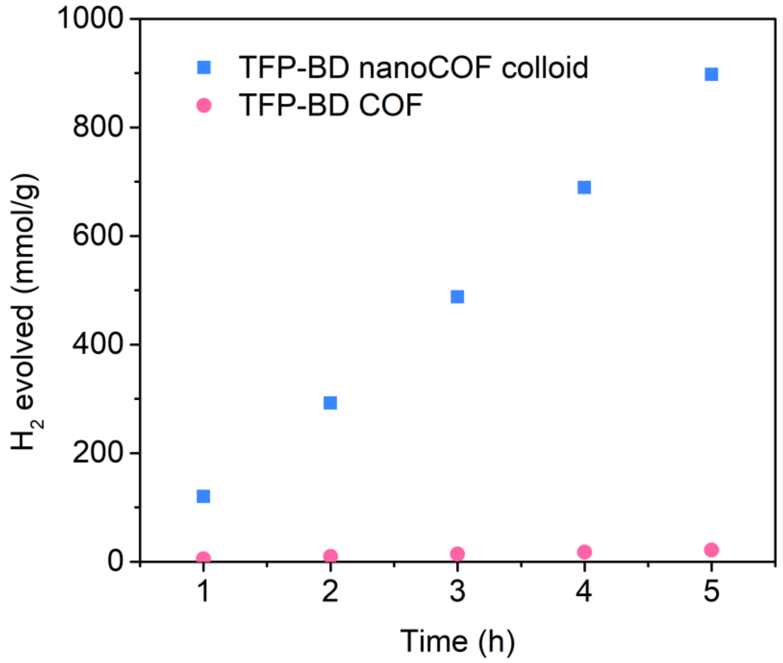

Supplementary Figure 26. Comparison of photocatalytic H_2 evolution of TFP-BD nano-COF with bulk TFP-BD COF over 5 h. Conditions for nano-COF: 0.5 mL TFP-BD nano-COF in 24.5 mL water, diluted H_2PtCl_6 solution as a platinum precursor (15 wt % *vs* photocatalysts), 880 mg AA (overall concentration is 0.2 M), $\lambda > 420$ nm; conditions for bulk COFs: 5 mg of bulk COF catalyst in water, diluted H_2PtCl_6 solution as a platinum precursor (4 wt % *vs* photocatalysts), 0.2 M AA (25 mL), $\lambda > 420$ nm.

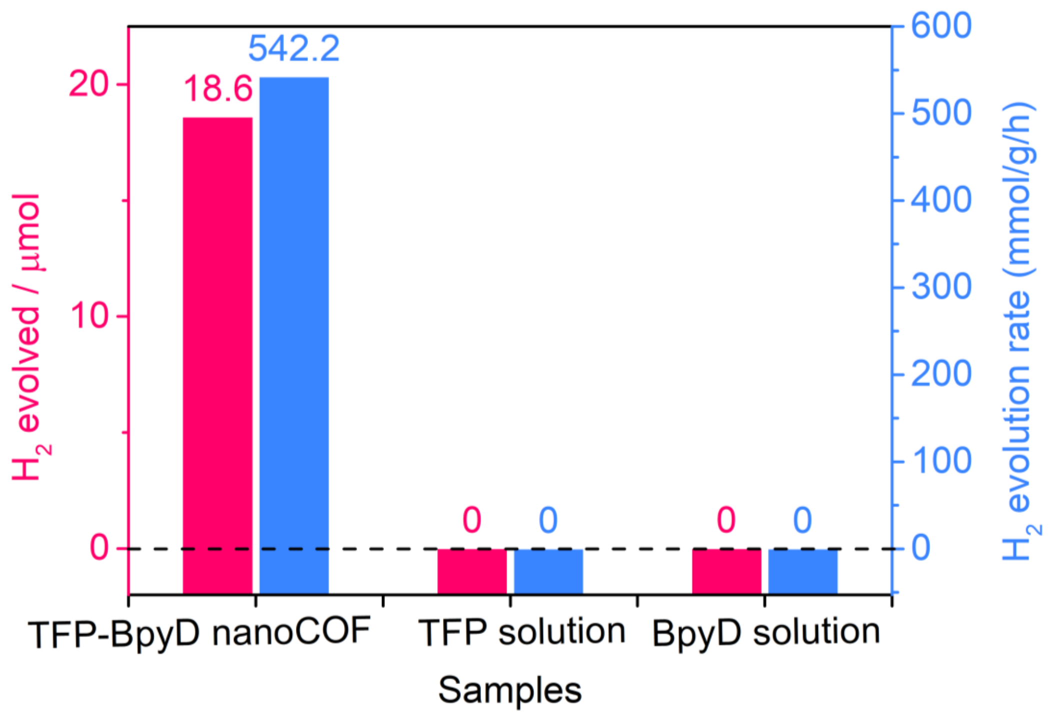

Supplementary Figure 27. Photocatalytic H_2 production for TFP-BpyD nano-COF with control samples (monomer solutions). Reaction conditions: TFP-BpyD nano-COF colloid (0.05 mL), TFP solution (0.05 mL) or BpyD solution (0.05 mL) in 0.2 M AA solution (5 mL total volume), 15 wt % Pt *vs* photocatalysts, 1 h illumination (Oriel Solar Simulator, 1.0 sun).

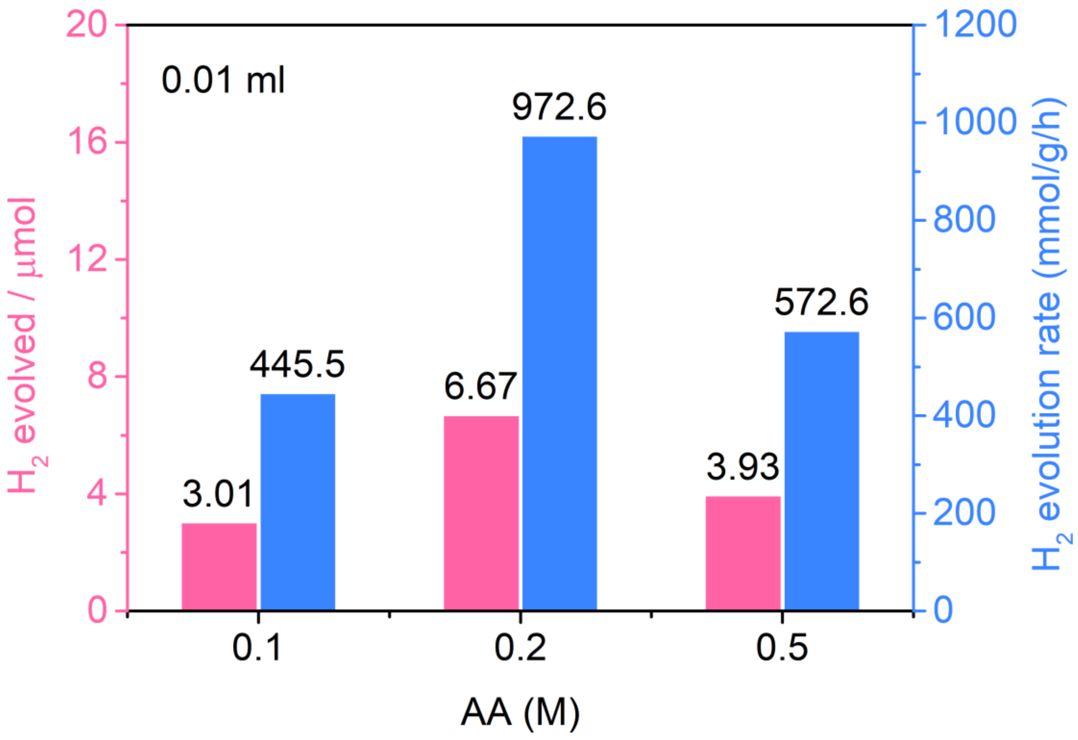

Supplementary Figure 28. TFP-BpyD nano-COF for sacrificial photocatalytic H_2 evolution. Condition: TFP-BpyD nano-COF (0.01 mL) in 0.1, 0.2 or 0.5 M AA solution (5 mL total volume), 15 wt % Pt *vs* photocatalysts, 2 h illumination (Oriel Solar Simulator, 1.0 sun). Blue bars: mass normalized H_2 evolution rate; pink bars: absolute H_2 amount evolved.

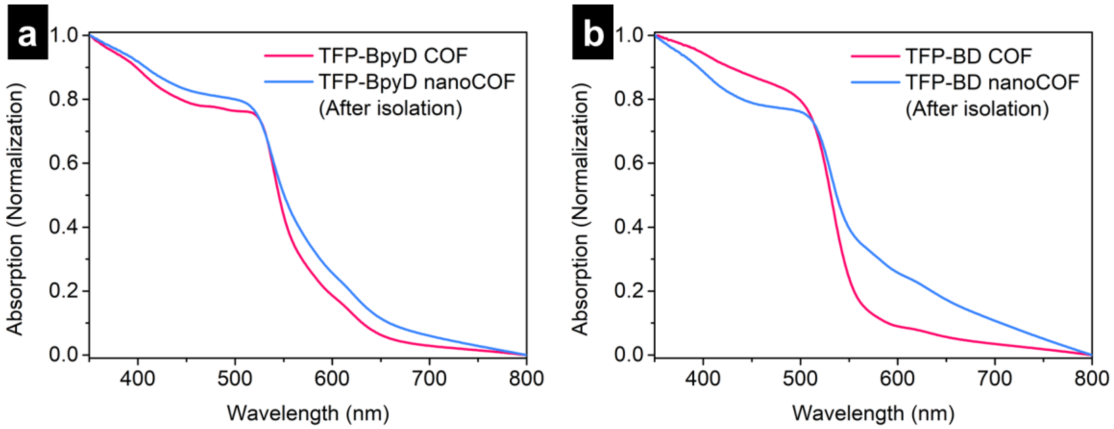

Supplementary Figure 29. Solid-state UV-vis spectra of COFs (normalized absorption units). (a) TFP-BpyD COF and isolated TFP-BpyD nano-COF. (b) TFP-BD COF and isolated TFP-BD nano-COF.

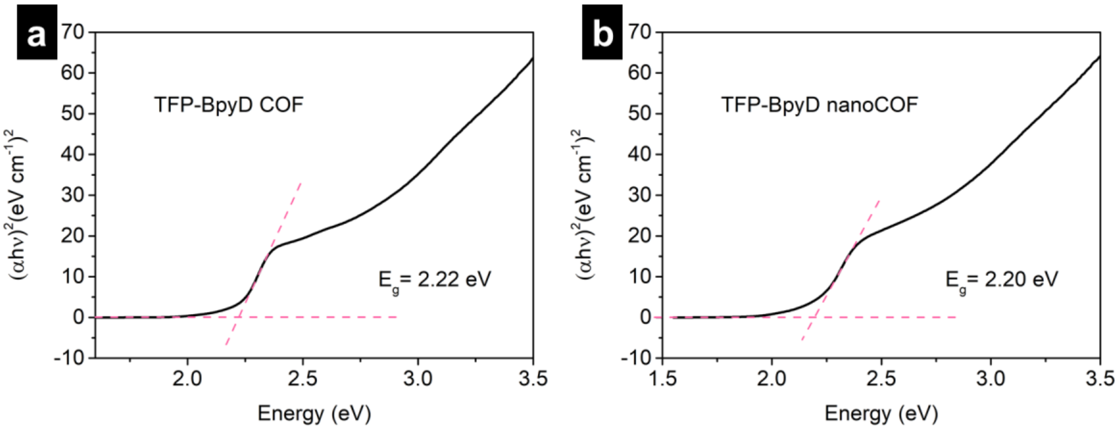

Supplementary Figure 30. Kubelka-Munk-transformed reflectance spectra and calculated optical gaps. (a) TFP-BpyD COF. (b) TFP-BpyD nano-COF.

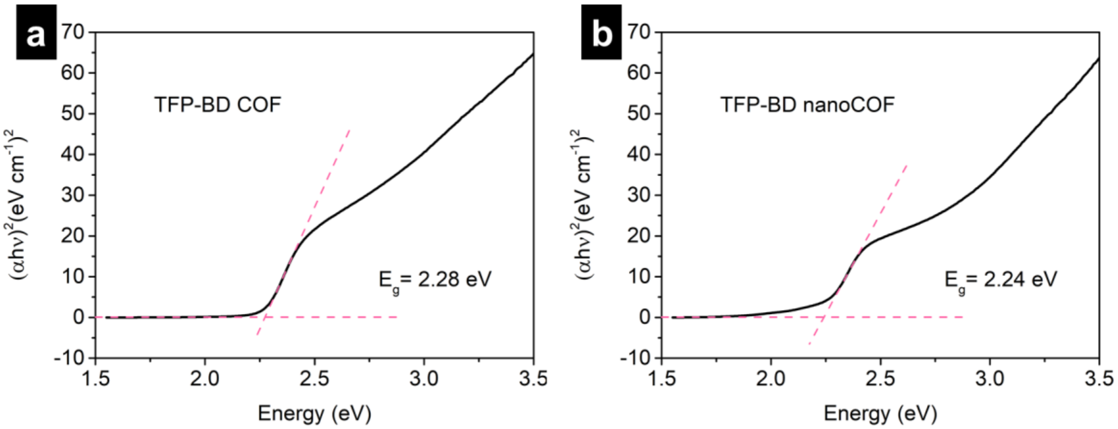

Supplementary Figure 31. Kubelka-Munk-transformed reflectance spectra and calculated optical gaps. (a) TFP-BD COF. (b) TFP-BD nano-COF.

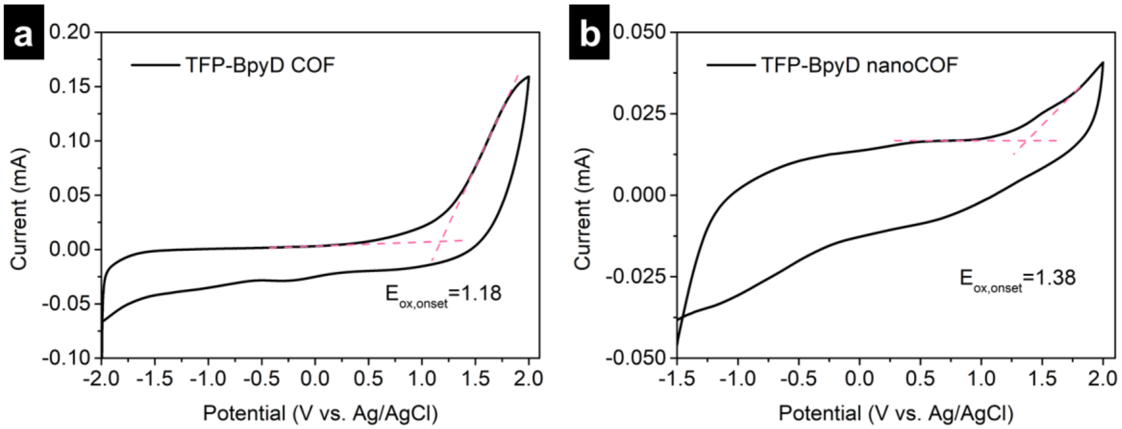

Supplementary Figure 32. CV curves of COFs. Oxidation onset potential of (a) TFP-BpyD COF and (b) solid TFP-BpyD nano-COF as measured by cyclic voltammetry at a scan rate of 50 mV/s.

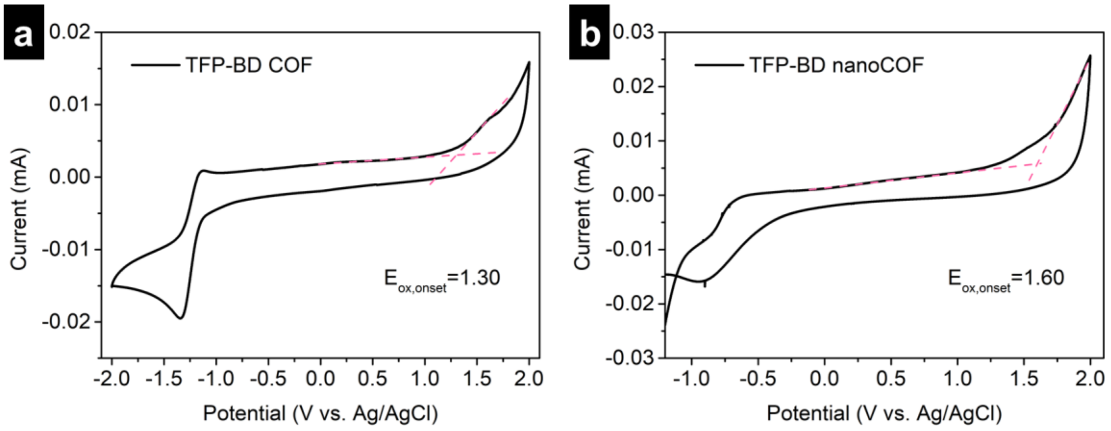

Supplementary Figure 33. CV curves of COFs. Oxidation onset potential of TFP-BD COF (a) and solid TFP-BD nano-COF (b) as measured by cyclic voltammetry at a scan rate of 50 mV/s.

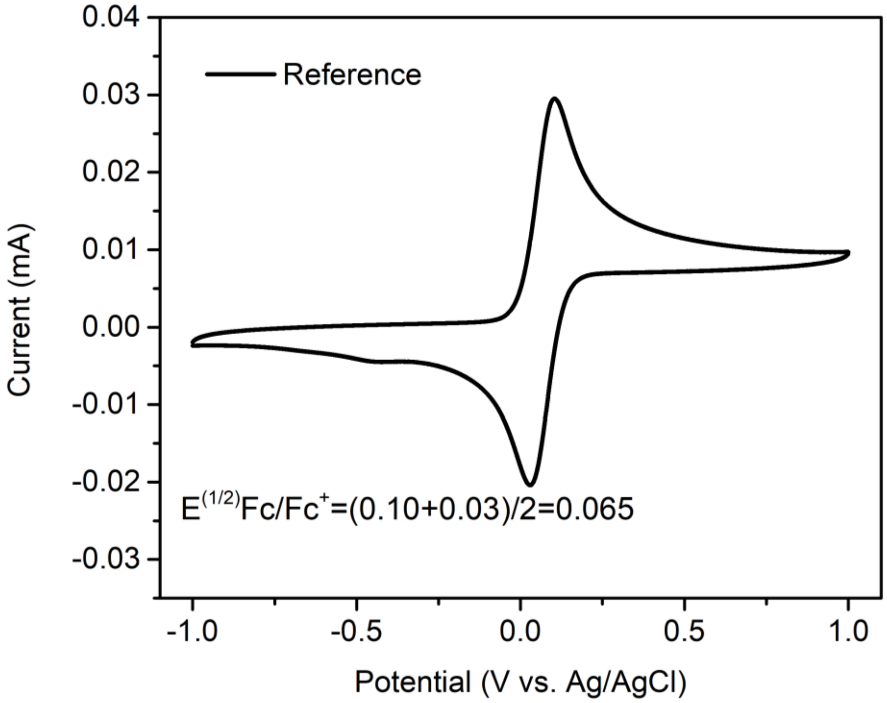

Supplementary Figure 34. CV curves of reference. Ferrocenium/ferrocene (Fc/Fc^+) redox couple used as an external potential reference.

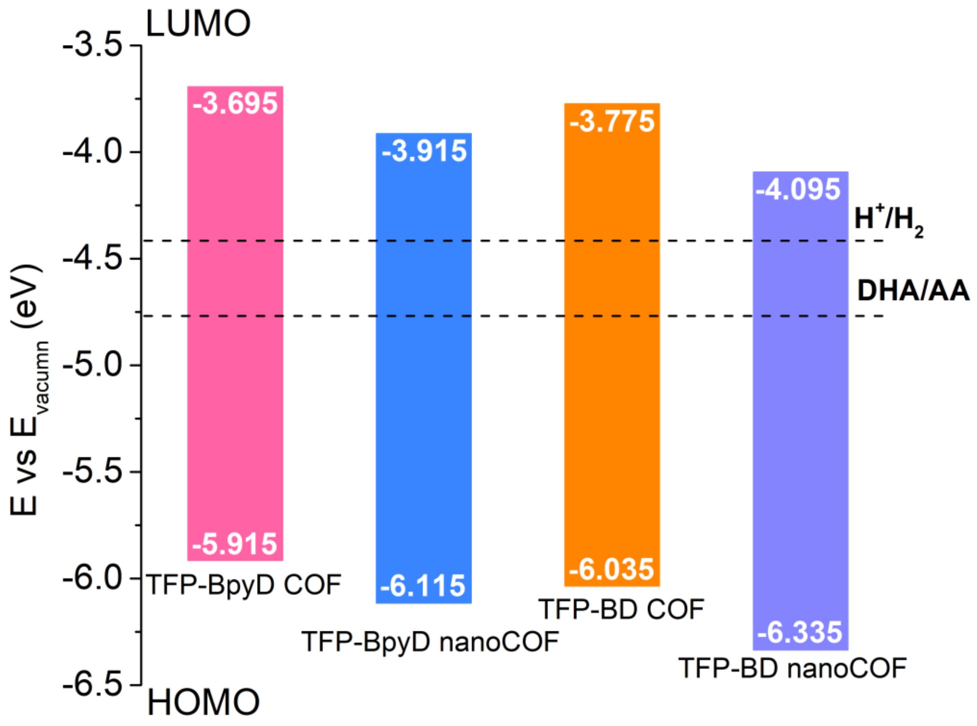

Supplementary Figure 35. Highest occupied and lowest unoccupied molecular orbital energy levels of TFP-BpyD COF, TFP-BpyD nano-COF, TFP-BD COF and TFP-BD nano-COF compared to the proton reduction potential (H^+/H_2). The band gaps were based on experimental measurements (Supplementary Figures 29–34), and the calculated potential for the two-hole oxidation of ascorbic acid to dehydroascorbic acid in solution (DHA/AA)[1] as determined from CV and UV measurements (0 V versus SHE = −4.44 V versus vacuum).

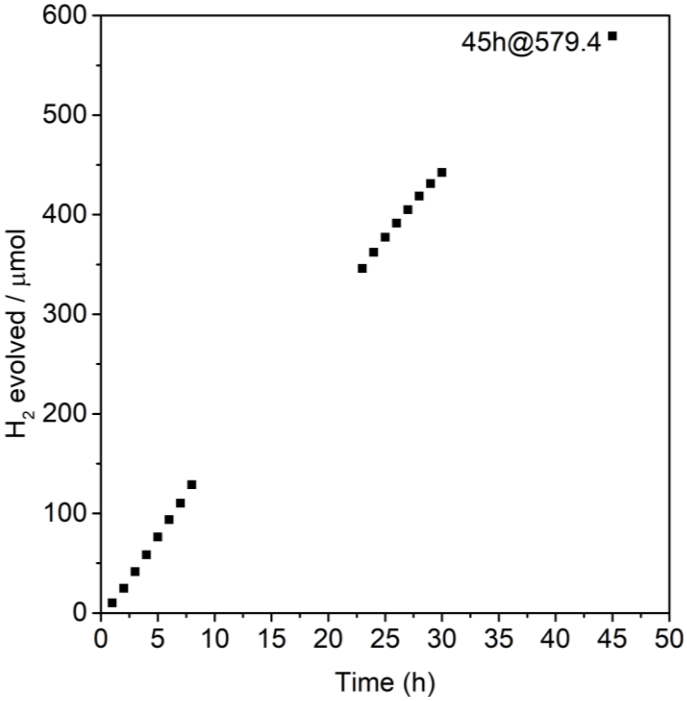

Supplementary Figure 36. Plot showing sacrificial photocatalytic H_2 evolution as a function of time for TFP-BD nano-COF over 45 h. Conditions: 0.5 mL TFP-BD nano-COF colloid in 24.5 mL water, diluted H_2PtCl_6 solution as a platinum precursor (15 wt% *vs* photocatalysts), 880 mg AA (overall concentration is 0.2 M), $\lambda > 420$ nm.

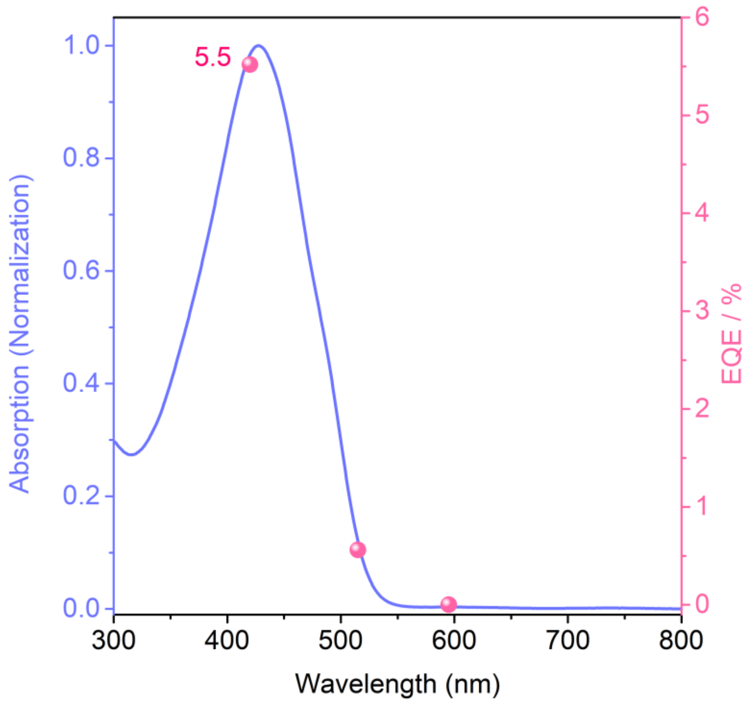

Supplementary Figure 37. Overlay of the UV/vis absorption spectrum for TFP-BD nano-COF with external quantum efficiency (EQE) at three different incident light wavelengths.

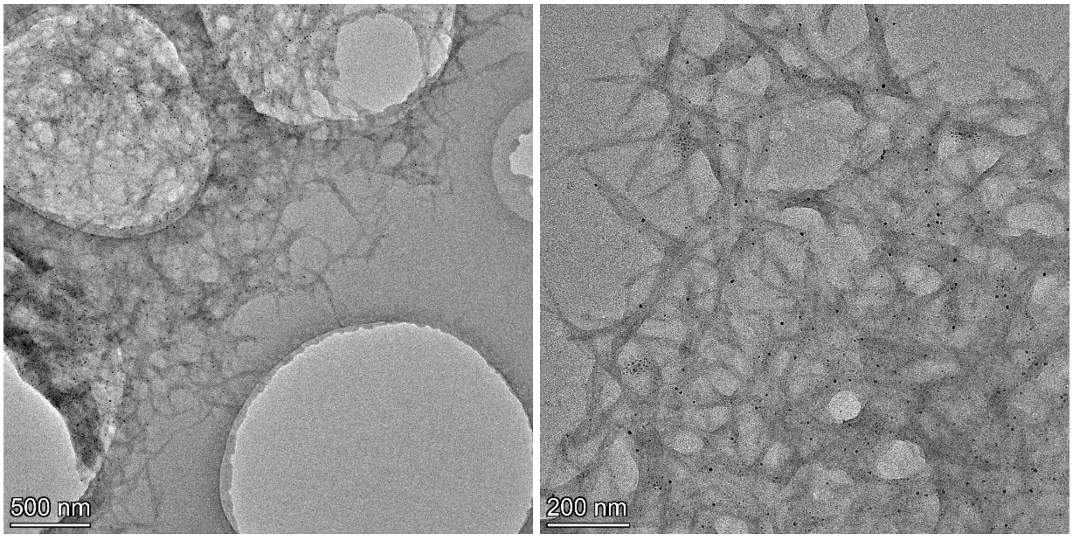

Supplementary Figure 38. TEM images for TFP-BpyD nano-COF decorated with photo-deposited Pt co-catalyst (dark / black spots). These images showed Pt nanoparticle were distributed uniformly on the nano-COFs.

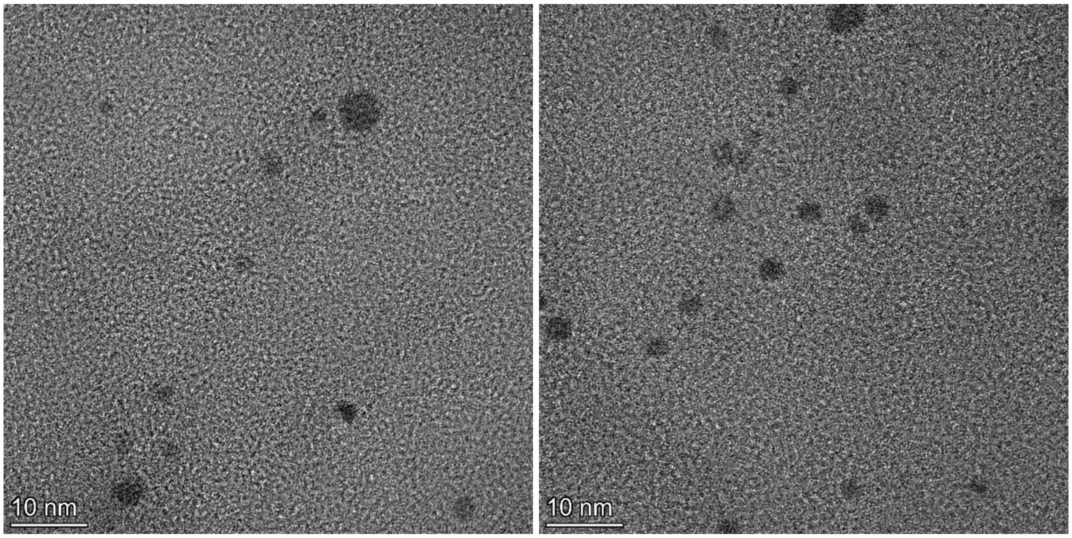

Supplementary Figure 39. TEM images for photo-deposited Pt co-catalyst on TFP-BpyD nano-COF (dark / black spots). These images showed Pt nanoparticle were distributed uniformly on the nano-COFs.

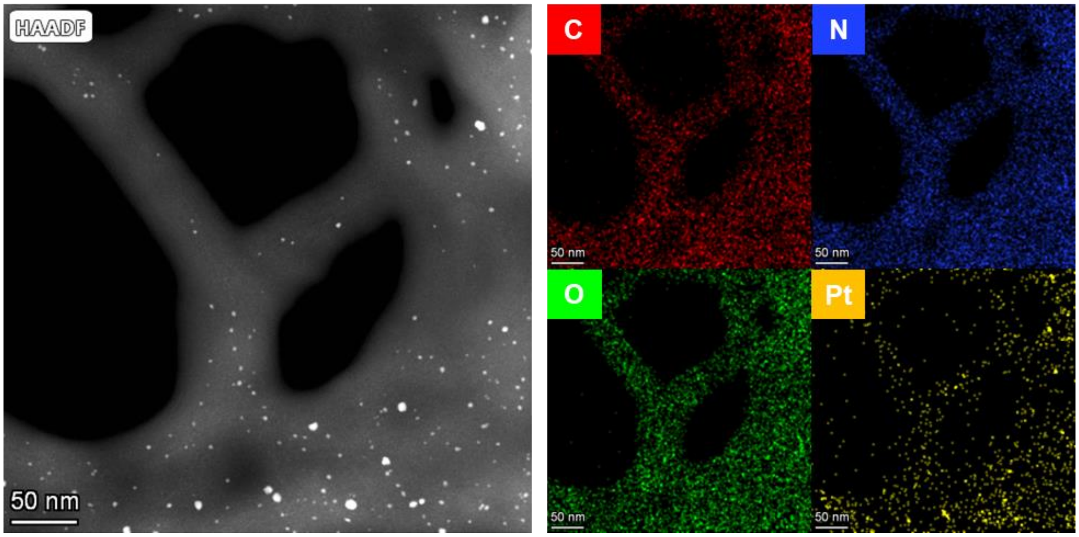

Supplementary Figure 40. High-angle annular dark field scanning transmission electron microscopy (HAADF-STEM) images and elemental mapping for TFP-BpyD nano-COF decorated with photo-deposited Pt co-catalyst. These images showed Pt nanoparticle were distributed uniformly on the nano-COFs.

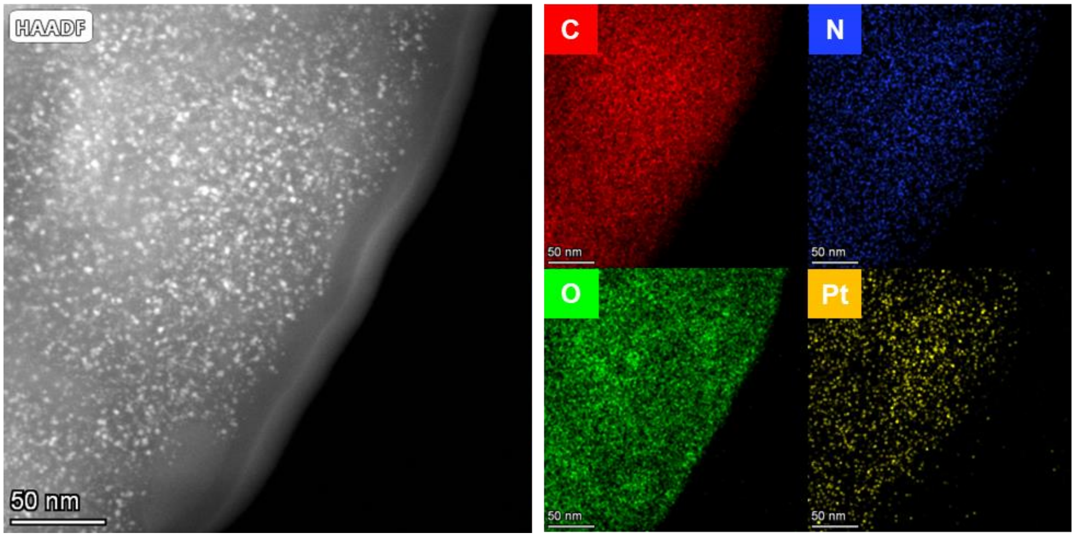

Supplementary Figure 41. High angle annular dark field scanning transmission electron microscopy (HAADF-STEM) images and elemental mapping for TFP-BD nano-COF decorated with photo-deposited Pt co-catalyst. These images showed Pt nanoparticle were distributed uniformly on the nano-COFs.

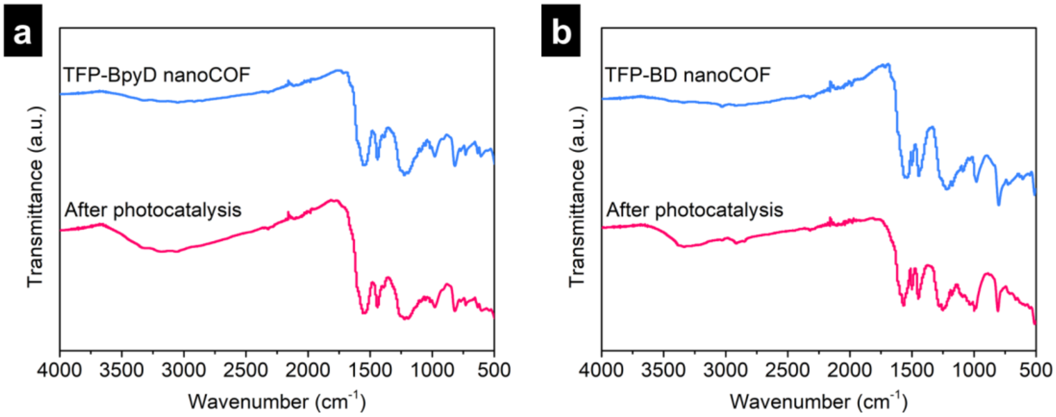

Supplementary Figure 42. **FT-IR spectra of nano-COFs before and after photocatalysis.** (a) TFP-BpyD nano-COF. (b) TFP-BD nano-COF.

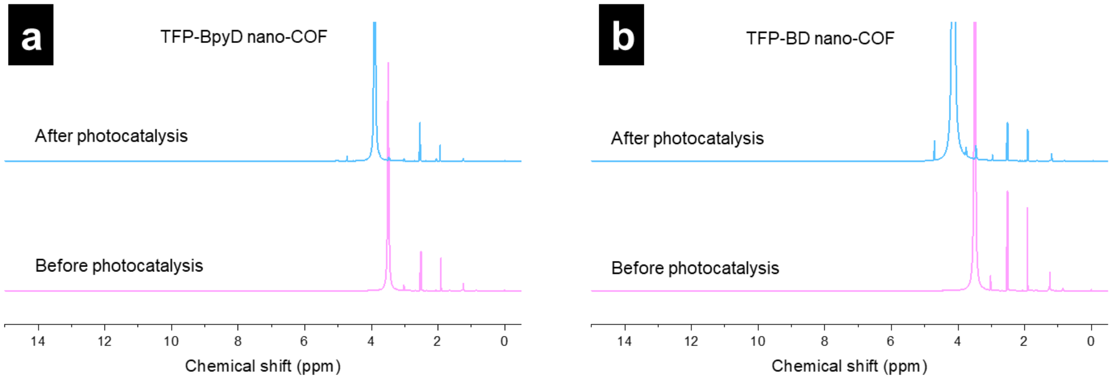

Supplementary Figure 43. NMR spectra of nano-COFs before and after photocatalysis. (a) TFP-BpyD nano-COF. (b) TFP-BD nano-COF. No monomer signals were detected. The shift for water signals (~3.33 ppm) after photocatalysis was due to the presence of AA.

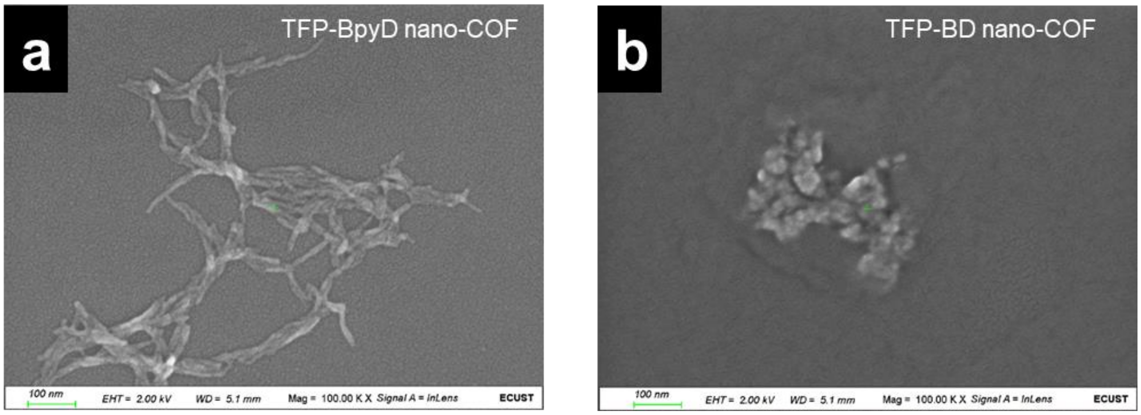

Supplementary Figure 44. SEM images of nano-COFs after photocatalysis. (a) TFP-BpyD nano-COF. (b) TFP-BD nano-COF.

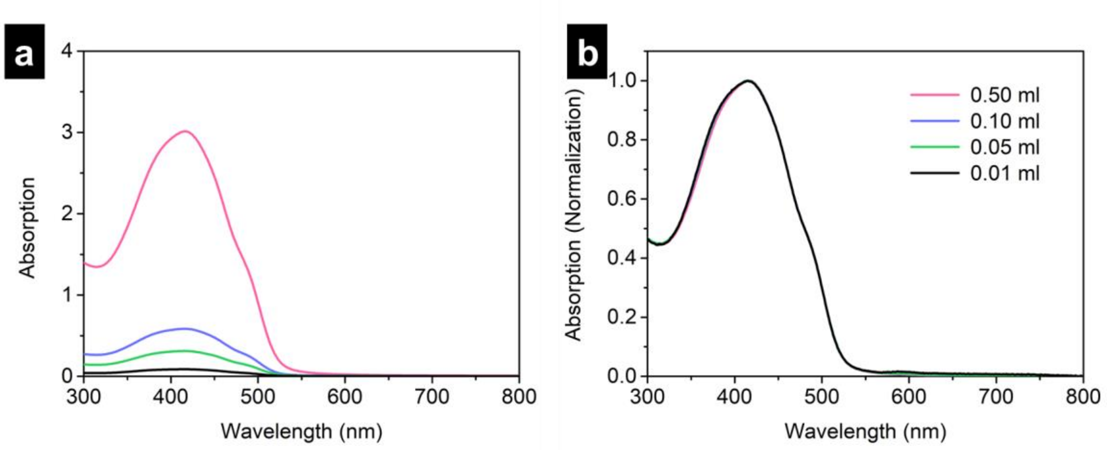

Supplementary Figure 45. UV-vis spectra of TFP-BpyD nano-COF at different concentrations before and after normalization. (a) Before and (b) after normalization. Conditions: 0.01, 0.05, 0.10 and 0.50 mL of TFP-BpyD nano-COF stock colloid solution were diluted by water to a total volume of 5 mL.

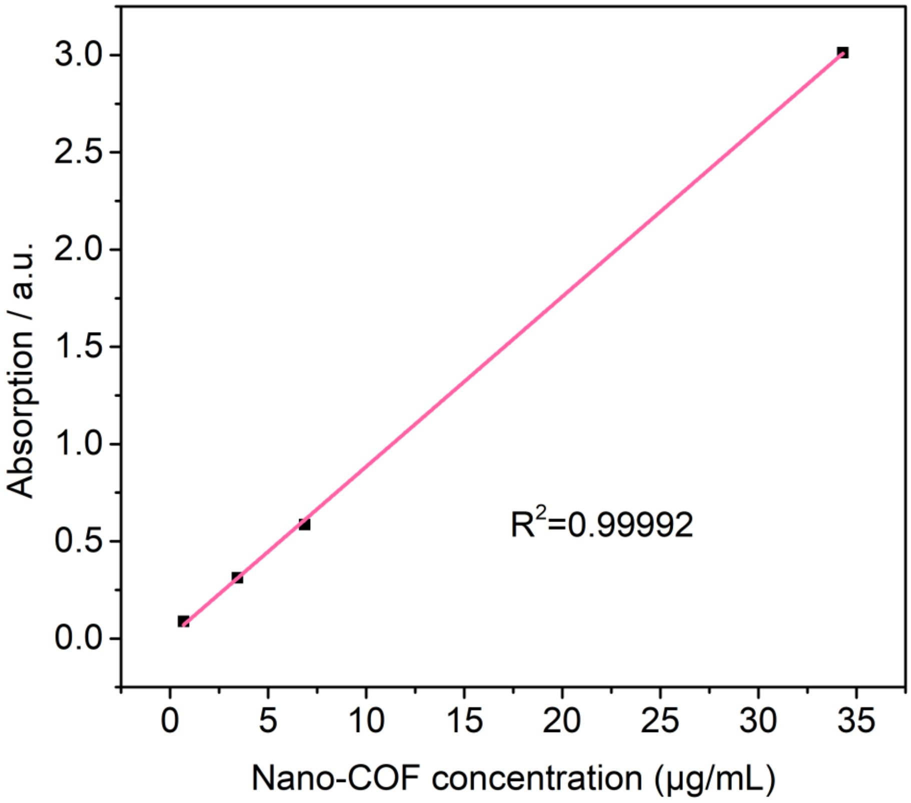

Supplementary Figure 46. Calibration curve for UV-vis spectra of TFP-BpyD nano-COF solutions using the absorption peak at 416 nm. Conditions: 0.01, 0.05, 0.10 and 0.50 mL TFP-BpyD nano-COF were diluted by water to a total volume of 5 mL.

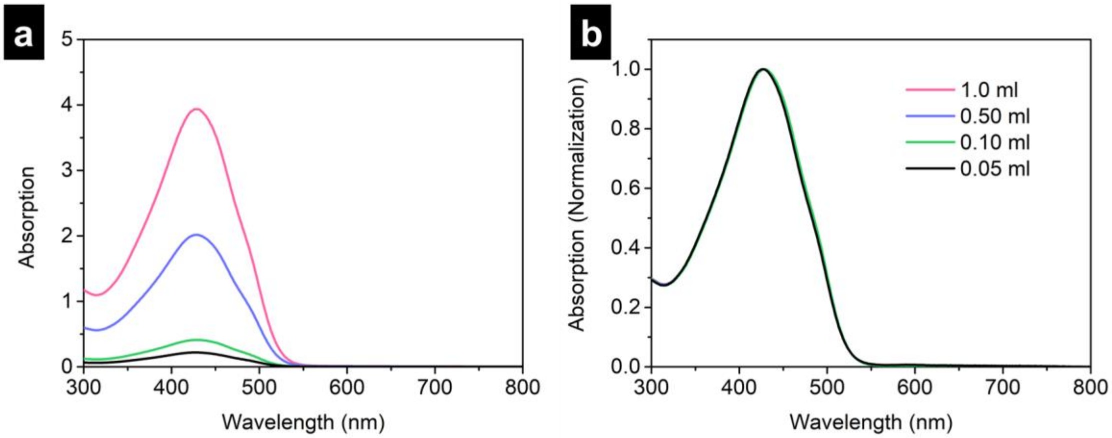

Supplementary Figure 47. UV spectra of TFP-BD nano-COF at different concentrations before and after normalization. (a) Before and (b) after normalization. Conditions: 0.05, 0.10, 0.50 and 1.0 mL TFP-BD nano-COF were diluted by water to a total volume of 5 mL.

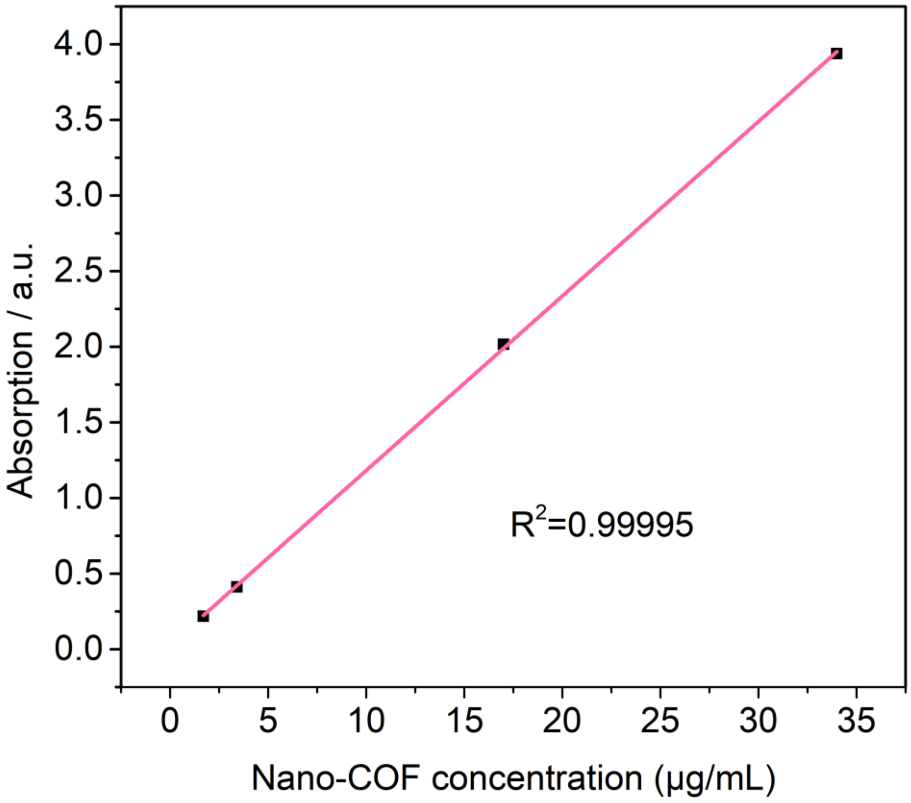

Supplementary Figure 48. Calibration curve for UV-vis spectra of TFP-BD nano-COF solutions using the absorption peak of 416 nm. Conditions: 0.05, 0.10, 0.50 and 1.0 mL TFP-BD nano-COF were diluted by water to a total volume of 5 mL.

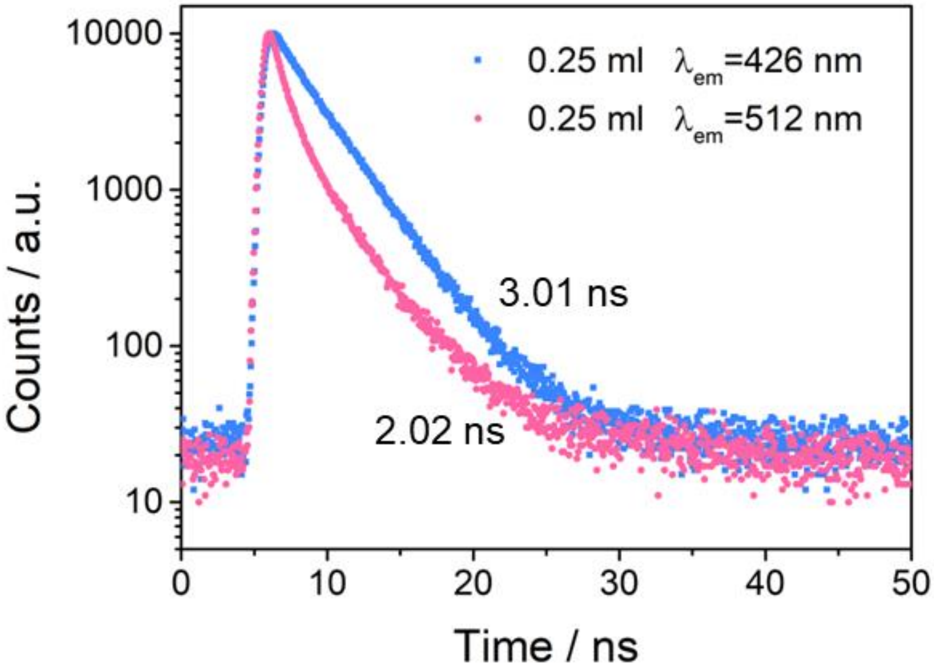

Supplementary Figure 49. TCSPC measurements of TFP-BpyD nano-COF with different emission wavelengths. Conditions: 0.25 mL TFP-BpyD nano-COF were diluted by water to a total volume of 5 mL.

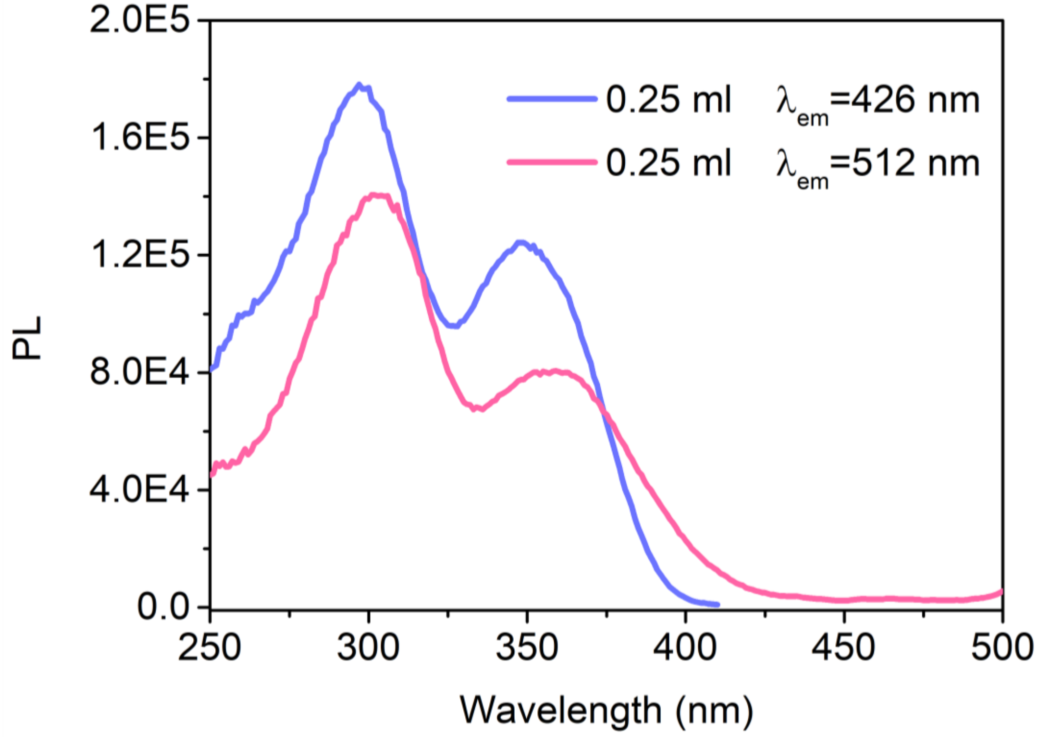

Supplementary Figure 50. Photoluminescence excitation spectra of TFP-BpyD nano-COF with different emission wavelengths. Conditions: 0.25 mL TFP-BpyD nano-COF were diluted by water to a total volume of 5 mL.

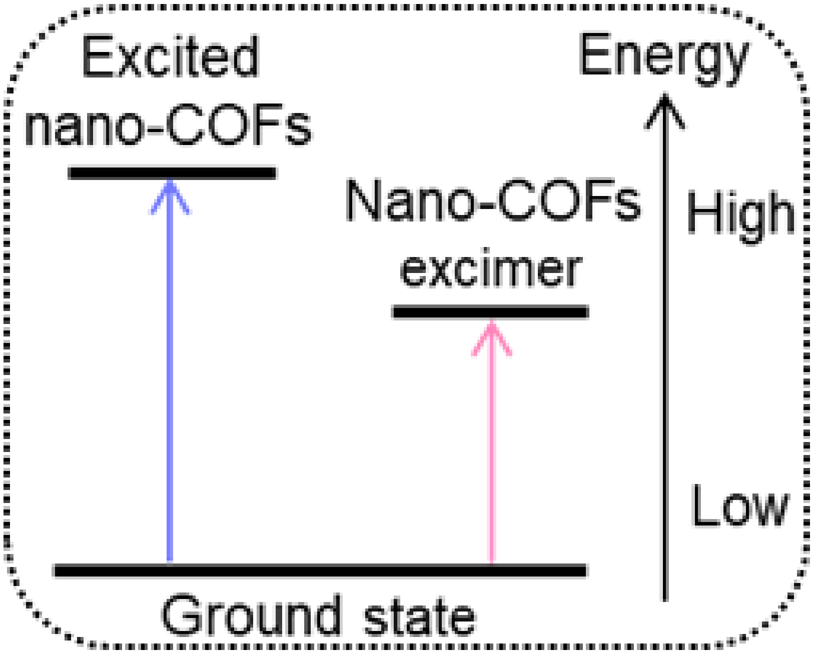

Supplementary Figure 51. Scheme for nano-COF excimer formation.

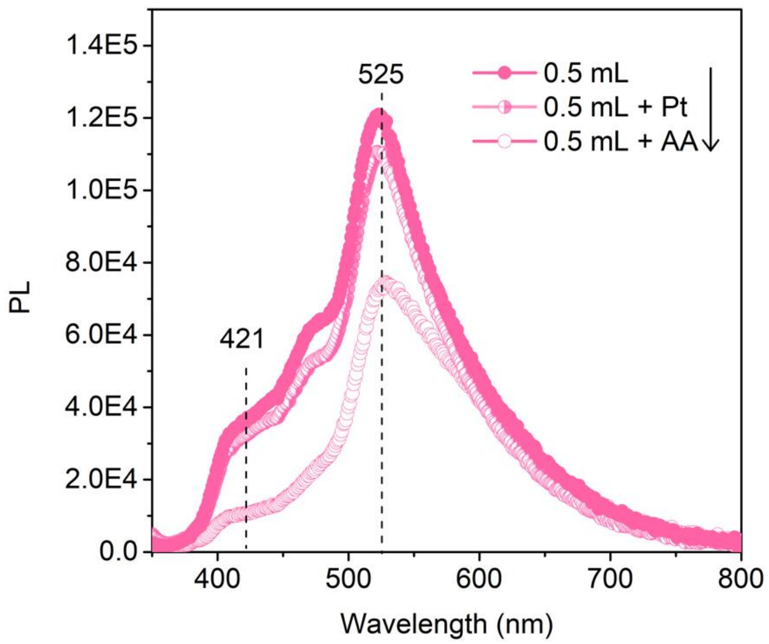

Supplementary Figure 52. Photoluminescence (PL) spectra of TFP-BpyD nano-COF with addition of (i) AA or (ii) Pt. Conditions: (i) 0.5 mL TFP-BpyD nano-COF colloid solution was diluted by 0.2 M AA (0.5 mL); (ii) 0.5 mL TFP-BpyD nano-COF colloid was diluted by water with Pt co-catalyst (15 wt% *vs* photocatalysts). The total volume was made up to 5 mL. To allow comparison at the same concentration, the 0.5 mL TFP-BpyD nano-COF colloid (solid symbols in plot) was diluted by water (0.5 mL).

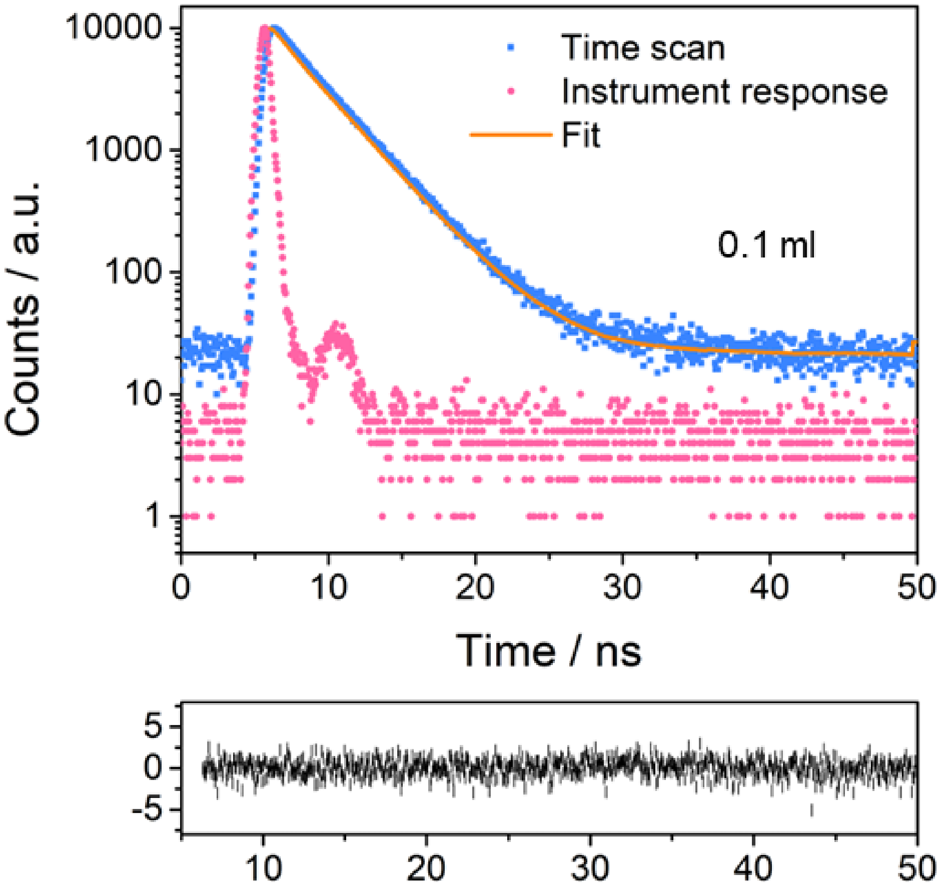

Supplementary Figure 53. TCSPC measurements of TFP-BpyD nano-COF (0.1 mL TFP-BpyD nano-COF was diluted by water to a total volume of 5 mL). The sample was excited with a λ_{ex} = 371 nm laser. The yellow line represents the fit and the black line are the weighted residuals of the fit.

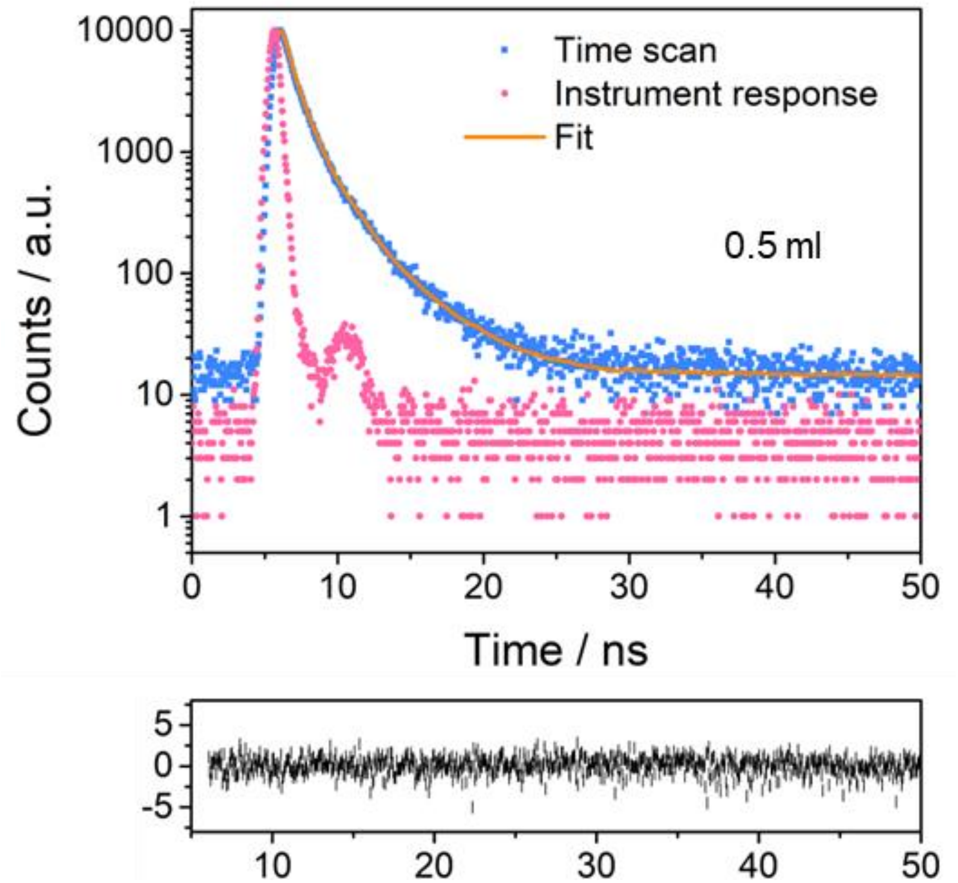

Supplementary Figure 54. TCSPC measurements of TFP-BpyD nano-COF (0.5 mL TFP-BpyD nano-COF was diluted by water to a total volume of 5 mL). The sample was excited with a λ_{ex} = 371 nm laser. The yellow line represents the fit and the black line are the weighted residuals of the fit.

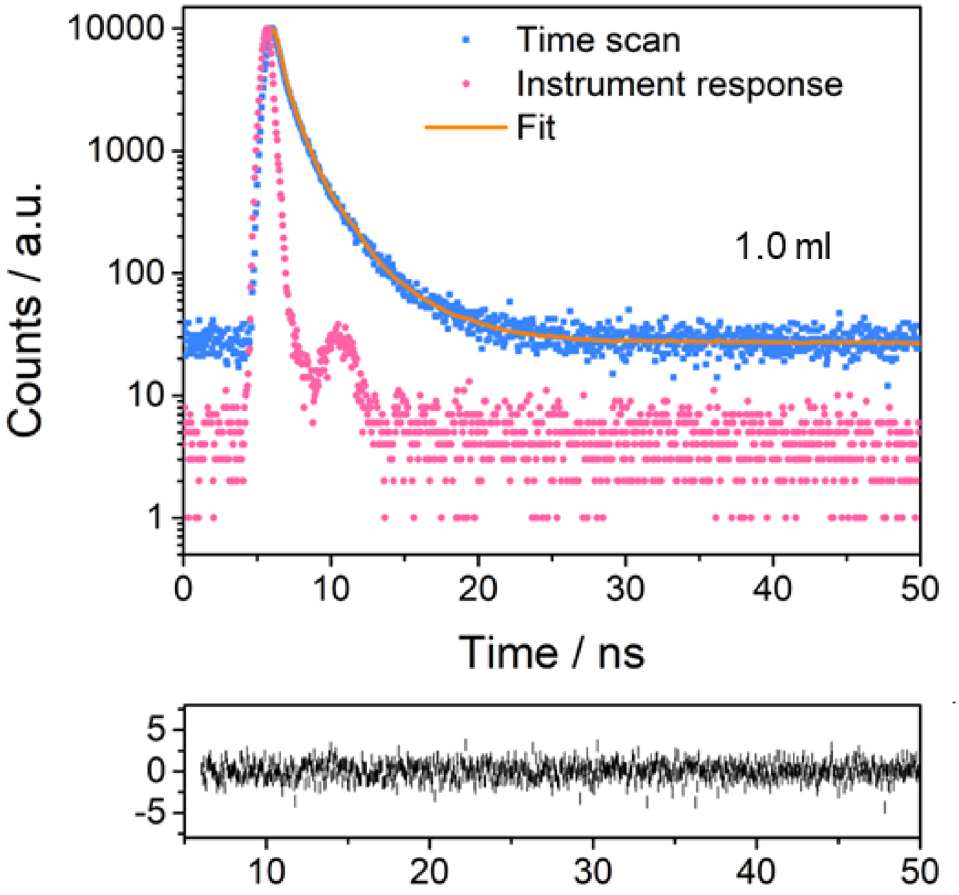

Supplementary Figure 55. TCSPC measurements of TFP-BpyD nano-COF (1.0 mL TFP-BpyD nano-COF was diluted by water a total volume of 5 mL). The sample was excited with a λ_{ex} = 371 nm laser. The yellow line represents the fit and the black line are the weighted residuals of the fit.

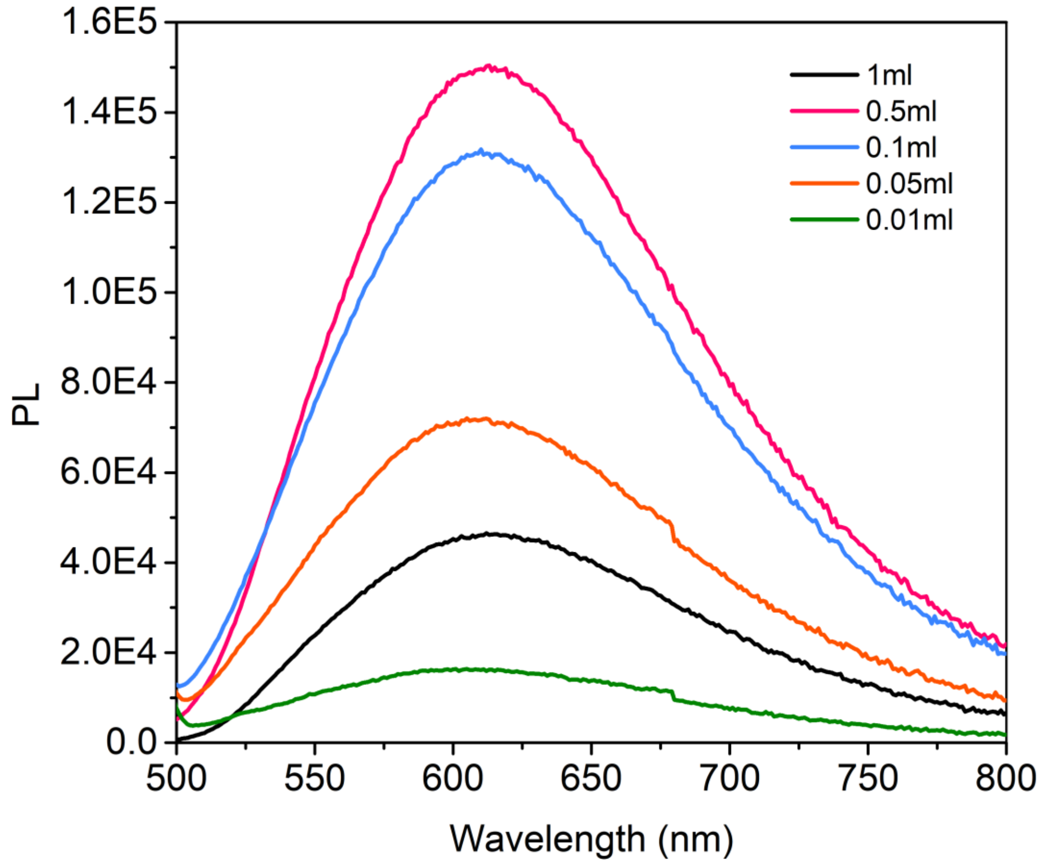

Supplementary Figure 56. Photoluminescence emission spectra of TFP-BD nano-COF colloids with different concentrations. Conditions: 0.01, 0.05, 0.10, 0.50 and 1.00 mL TFP-BD nano-COF colloids were diluted by water to a total volume of 5 mL.

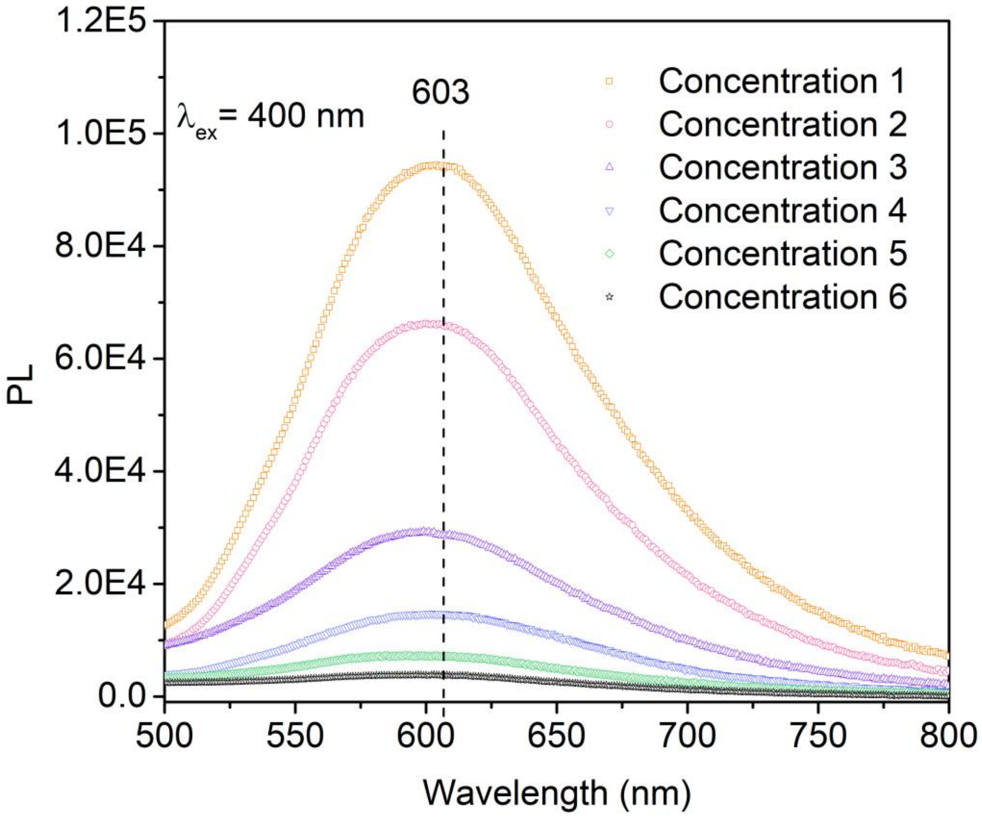

Supplementary Figure 57. Photoluminescence emission spectra of TFP-BpyD COF excited at 400 nm with different concentrations. Concentration 1: 0.0686 mg/mL; concentration 2: 0.0343 mg/mL; concentration 3: 0.0172 mg/mL; concentration 4: 0.00686 mg/mL; concentration 5: 0.00343 mg/mL; concentration 6: 0.000686 mg/mL. These concentrations are consistent with those using 1, 0.5, 0.25, 0.1, 0.05 and 0.01 mL of the TFP-BpyD nano-COF stock solution, respectively.

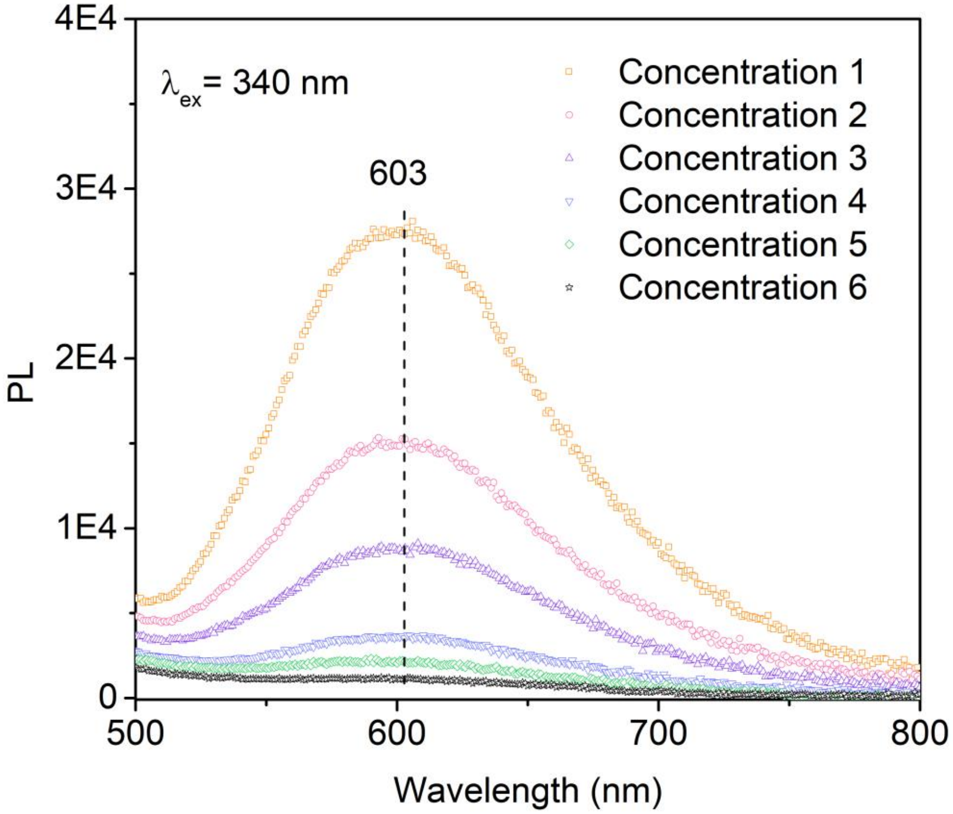

Supplementary Figure 58. Photoluminescence emission spectra of TFP-BpyD COF excited at 340 nm with different concentrations. Concentration 1: 0.0686 mg/mL; concentration 2: 0.0343 mg/mL; concentration 3: 0.0172 mg/mL; concentration 4: 0.00686 mg/mL; concentration 5: 0.00343 mg/mL; concentration 6: 0.000686 mg/mL. These concentrations are consistent with those using 1, 0.5, 0.25, 0.1, 0.05 and 0.01mL TFP-BpyD nano-COF.

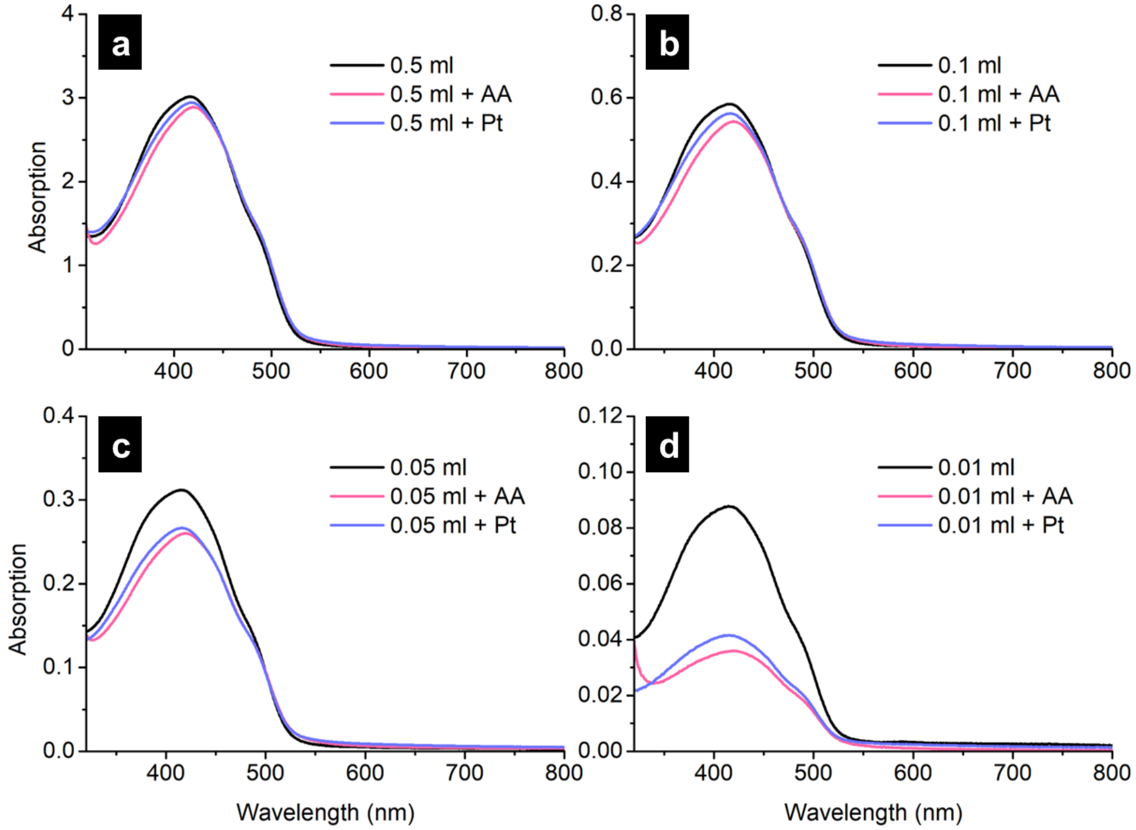

Supplementary Figure 59. UV spectra of TFP-BpyD nano-COF with ascorbic acid (AA) or co-catalyst (Pt) at different concentrations. (a) 0.5 mL, (b) 0.1 mL, (c) 0.05 mL and (d) 0.01 mL TFP-BpyD nano-COF were used, respectively. Conditions (without AA and Pt): 0.01, 0.05, 0.10 and 0.50 mL TFP-BpyD nano-COF were diluted by pure water to a total volume of 5 mL; Conditions (AA): 0.01, 0.05, 0.10 and 0.50 mL TFP-BpyD nano-COF were diluted by 0.2 M AA to a total volume of 5 mL; Conditions (Pt): 0.01, 0.05, 0.10 and 0.50 mL TFP-BpyD nano-COF were diluted by water to a total volume of 5 mL and 15 wt. % Pt based on the mass of COF was added.

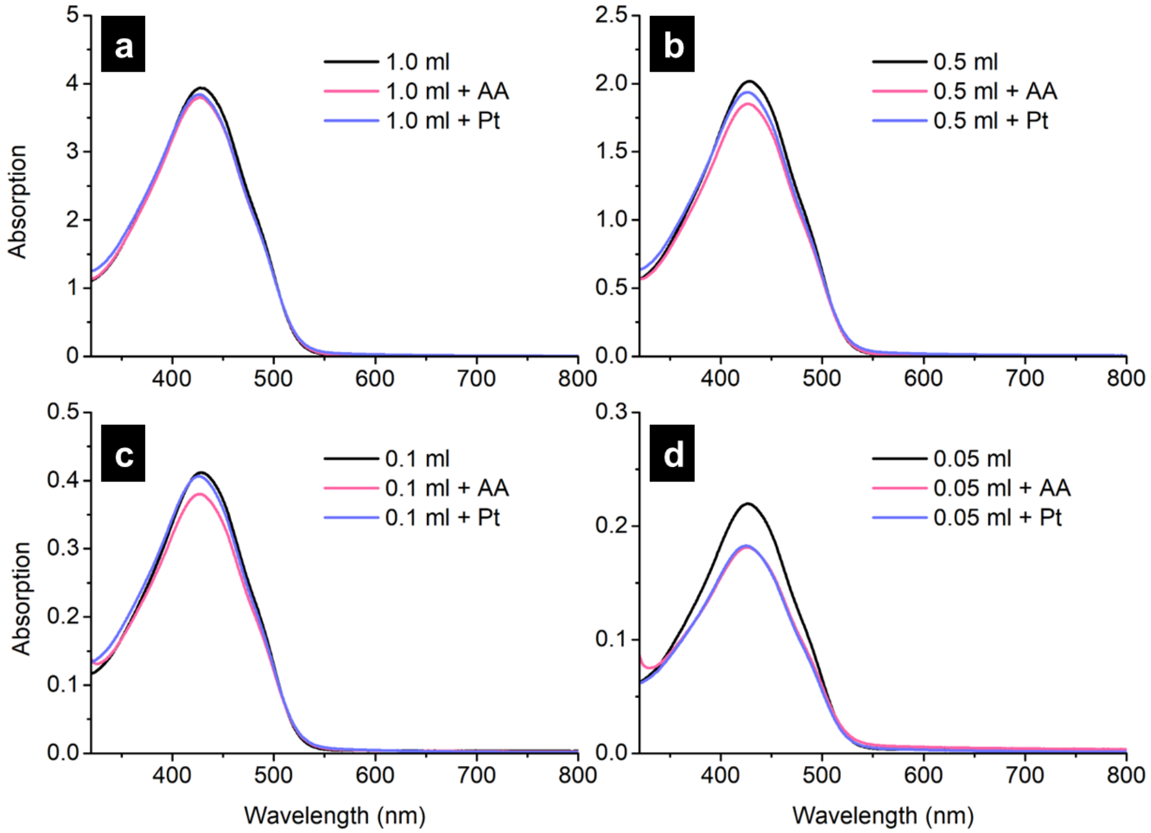

Supplementary Figure 60. UV spectra of TFP-BD nano-COF with ascorbic acid (AA) or co-catalyst (Pt) at different concentrations. (a) 1.0 mL, (b) 0.5 mL, (c) 0.1 mL and (d) 0.05 mL TFP-BD nano-COF were used, respectively. Conditions (without AA and Pt): 0.05, 0.1, 0.5 and 1.0 mL TFP-BD nano-COF were only diluted by water and the totally volume is 5 mL; Conditions (AA): 0.05, 0.1, 0.5 and 1.0 mL TFP-BD nano-COF were diluted by 0.2 M AA and the totally volume is 5 mL; Conditions (Pt): 0.05, 0.1, 0.5 and 1.0 mL TFP-BD nano-COF were diluted by water. The total volume was 5 mL and 15 wt % Pt based on the mass of COF was added.

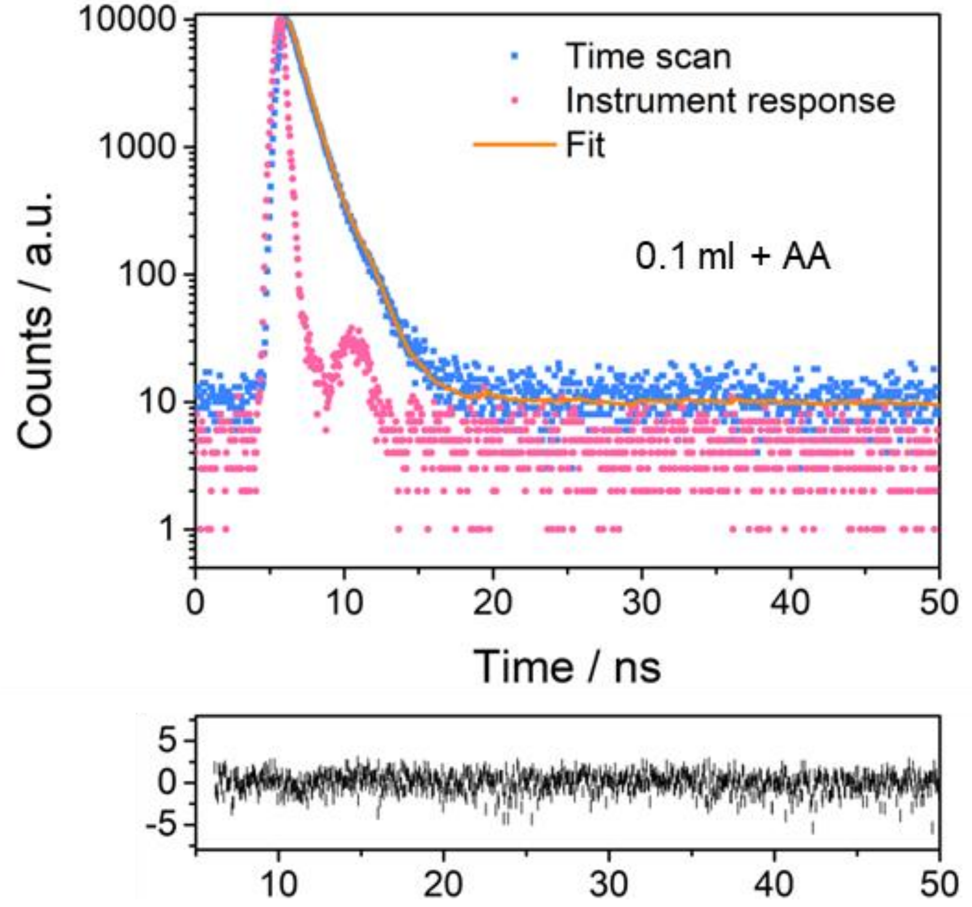

Supplementary Figure 61. TCSPC measurements of TFP-BpyD nano-COF (0.1 mL TFP-BpyD nano-COF was diluted by 0.2 M AA to 5 mL). The sample was excited with a λ_{ex} = 371 nm laser. The yellow line represents the fit and the black line are the weighted residuals of the fit.

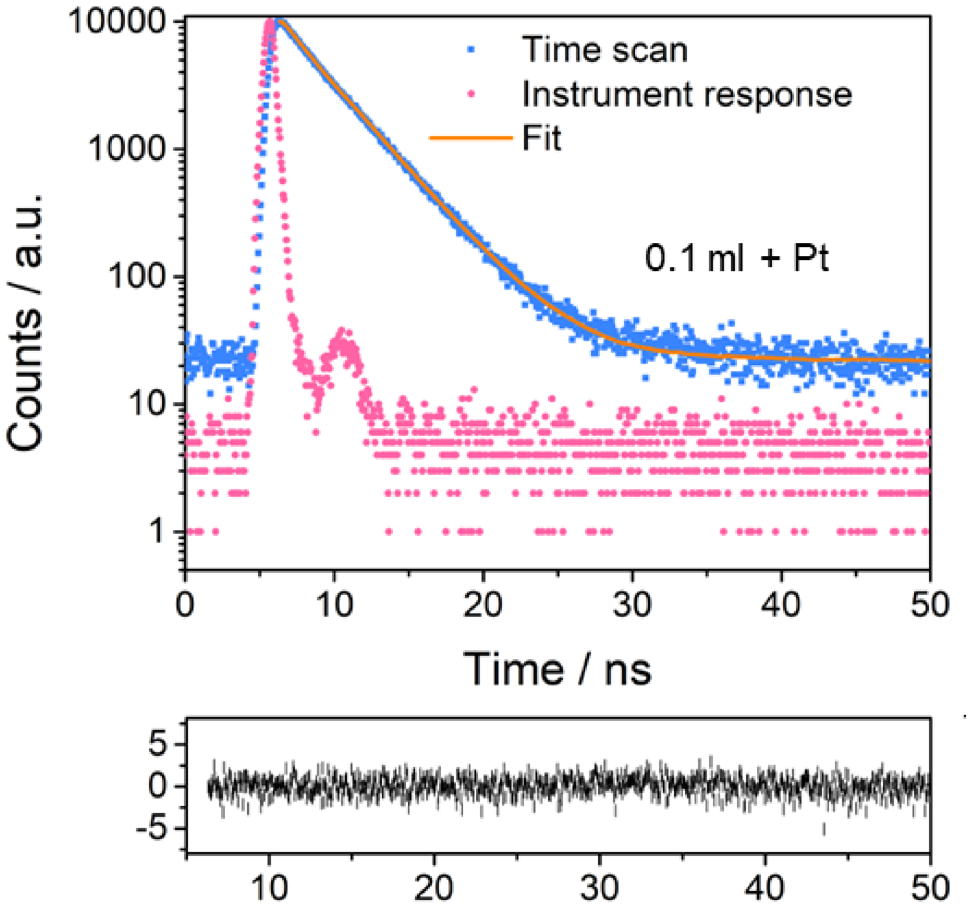

Supplementary Figure 62. TCSPC measurements of TFP-BpyD nano-COF (0.1 mL TFP-BpyD nano-COF was diluted by water to 5 mL and 15 wt% Pt was added). The sample was excited with a λ_{ex} = 371 nm laser. The yellow line represents the fit and the black line are the weighted residuals of the fit.

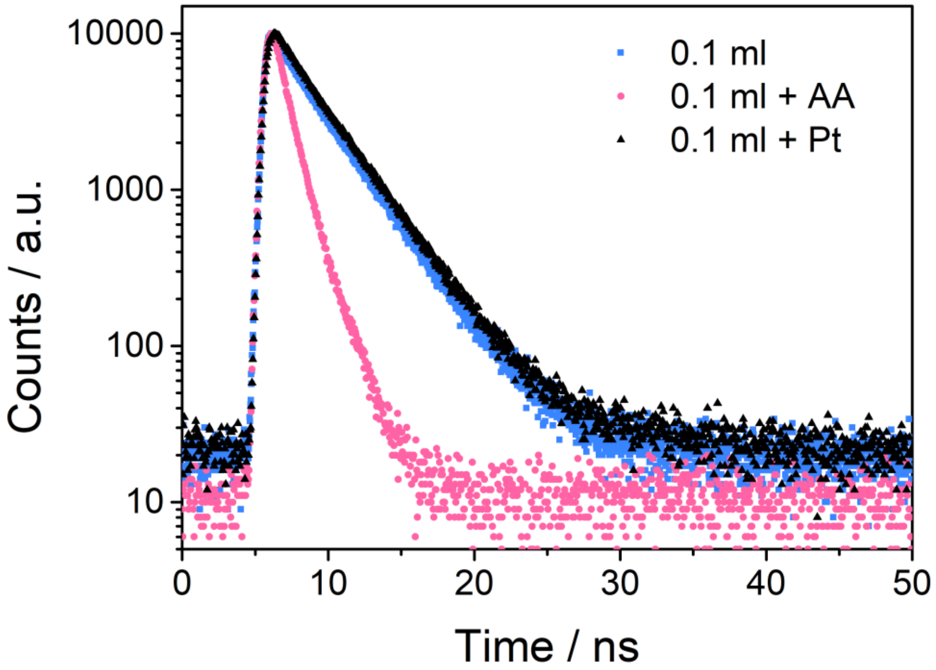

Supplementary Figure 63. Comparison of TCSPC measurements of TFP-BpyD nano-COF with different conditions. Conditions: 0.1 mL TFP-BpyD nano-COF were diluted by water, 0.2 M AA or water with 15 wt% Pt. The totally volume is 5 mL. The sample was excited with a λ_{ex} = 371 nm laser.

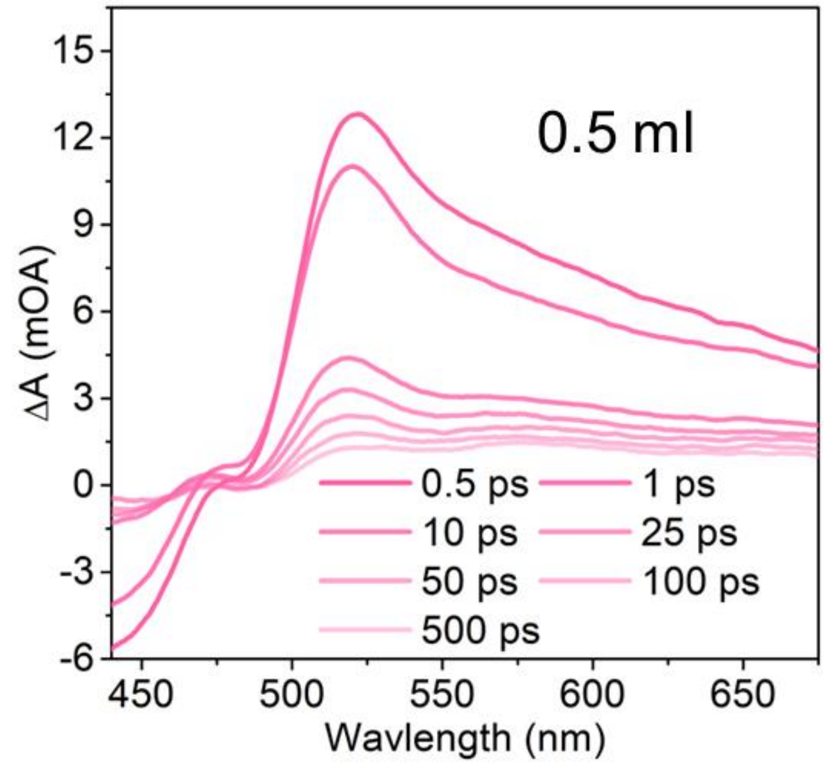

Supplementary Figure 64. TAS spectra of TFP-BpyD nano-COF. Conditions: 0.5 mL TFP-BpyD nano-COF was diluted by water to a total volume of 5 mL.

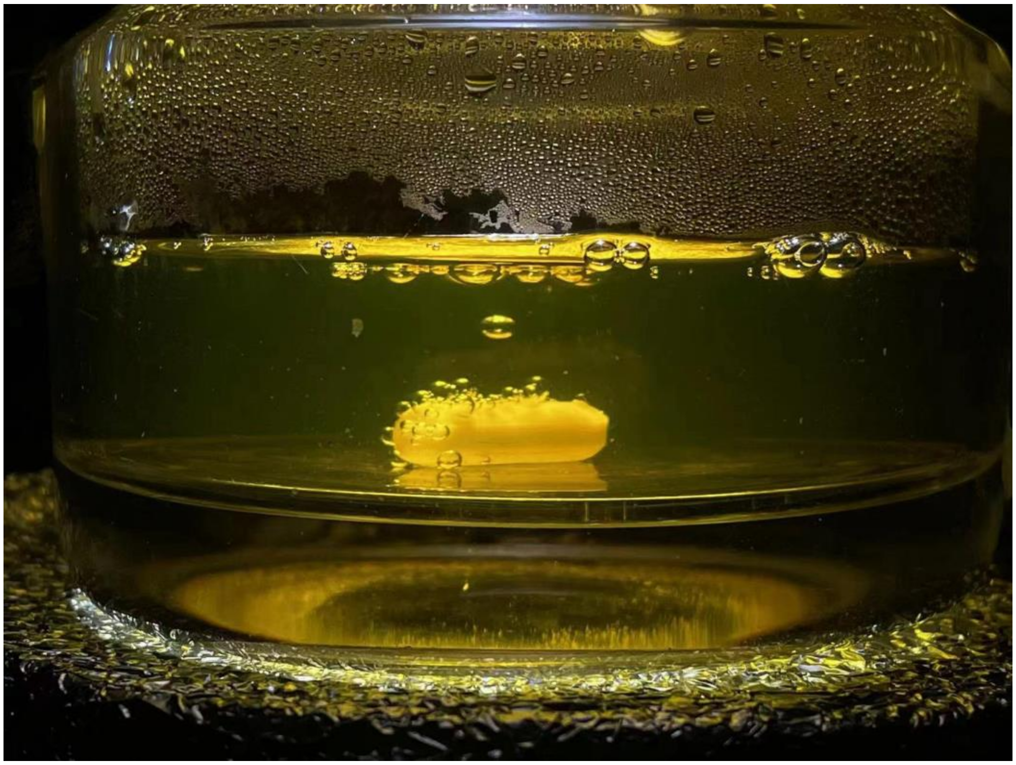

Supplementary Figure 65. Optical image of TFP-BpyD nano-COF for photocatalytic H_2 production. It showed H_2 bubbles produced by TFP-BpyD nano-COF.

Supplementary Table 1. Elemental analyses of the **COFs**.

Sample	C (wt %)		H (wt %)		N (wt %)	
	Calc.	Expt.	Calc.	Expt.	Calc.	Expt.
TFP-BD nano-COF (After isolation)	74.99	70.59	4.20	4.72	9.72	8.11
TFP-BD COF	74.99	70.90	4.20	4.88	9.72	8.27
TFP-BpyD nano-COF (After isolation)	66.20	65.07	3.47	4.46	19.30	15.95
TFP-BpyD COF	66.20	63.00	3.47	4.37	19.30	15.82

Calc. = calculated data, assuming idealized stoichiometry, infinite frameworks, and no physisorption of guests (*e.g.*, N_2, H_2O in the COF pores); note that none of these assumptions are satisfied in the real materials, and hence elemental analyses for these porous solids can differ significantly from the idealized, calculated values; Expt. = experimental results.

Supplementary Table 2. Summary of synthetic details for sonochemical preparation of sonoCOFs.

COFs	Amines	Aldehydes	Concentration of aqueous AcOH	Volume of aqueous AcOH (mL)	Sonication power (%)	Yield (%)
TFP-BpyD COF	BpyD 27.9 mg	TFP 21 mg	12 M	4	25	93
TFP-BD COF	BD 27.6 mg	TFP 21 mg	12 M	2	25	66

Supplementary Table 3. Summary of representative pristine COFs and other organic photocatalysts reported for sacrificial photocatalytic hydrogen evolution.

COFs	Band gap (eV)	Co-catalyst	Sacrificial agent	HER (mmol g^{-1} h^{-1})	AQY[h] (%)	Ref.
SonoCOF-3	2.46	Pt	AA	16.6[b]	3.71 (420 nm)[f]	2
SonoCOF-3	2.46	Pt	AA	24.5[c]	-	2
N_3-COF	2.6-2.7	Pt	TEOA	1.70	0.44 (450 nm)[d]	3
TP-COF	1.97	PVP-Pt	AA	8.42	0.4 (475 nm)[e]	4
PTP-COF	2.10	Pt	TEOA	0.08	0.87 (420nm)	5
N_2-COF	-	Co-1[a]	TEOA	0.78	0.16[d]	6
TTB-COF	2.8	Au	TEOA	0.15	-	7
TpDTz COF	2.07	NiME	TEOA	0.94	0.2 (400 nm)[e]	8
BT-TAPT-COF	2.35	Pt	AA	0.95	0.19 (410 nm)[f]	9
Py-HTP-BT-COF	2.25	Pt	AA	1.08	-	10
Py-FTP-BT-COF	2.34	Pt	AA	2.88	-	10
Py-ClTP-BT-COF	2.36	Pt	AA	8.88	8.45 (420 nm)[e]	10
g-$C_{40}N_3$-COF	2.36	Pt	TEOA	2.60	4.84 (420 nm)	11
TpPa-2-COF	2.52	Pt	LA	0.03	-	12
TpPa-2	2.07	Pt	SA	0.07	-	13
TpPa-COF-$(CH_3)_2$	2.06	Pt	SA	8.33	-	14
TP-BDDA	2.31	Pt	TEOA	0.32	1.8 (520 nm)[e]	15
TpPa-1-COF	2.02	Pt	SA	1.22	-	16
sp^2c-COF	1.9	Pt	TEOA	1.36	-	17
sp^2c-COF_{ERDN}	1.85	Pt	TEOA	2.12	0.48 (495 nm)	17
TFPT-COF	2.8	Pt	TEOA	1.97	2.2 (400 nm)[g]	18
FS-COF	1.85	Pt	AA	10.1	3.2 (420 nm)[f]	19
g-$C_{18}N_3$-COF	2.42	Pt	TEOA	0.29	1.06 (420 nm)	20
TpPa-1-COF	2.11	MoS_2	AA	5.59	0.76 (420 nm)[e]	21
TpPa-1	2.11	Pt	AA	5.48	-	21
BtCOF150	2.10	Pt	TEOA	0.75	0.2 (420 nm)	22
A-TEBPY-COF	1.94	Pt	TEOA	0.10	-	23
NTU-BDA-THTA	2.09	Pt	AA	1.13	-	24
PyTz-COF	2.20	Pt	AA	2.07	-	25
NKCOF-108	1.82	Pt	AA	11.6	2.96 (520 nm)	26
RC-COF-1	-	Pt	AA	27.9	6.4 (420 nm)	27
PY-DHBD-COF	2.28	Pt	AA	42.4	6.4 (420 nm)	28
Tz-COF-3	1.96	Pt	AA	43.2	6.9 (420 nm)	29
COF-JLU100	1.95	Pt	TEOA	107.4	5.1 (450 nm)	30
$[Mo_3S_{13}]^{2-}$@ZnP-Pz-PEO-COF	-	-	AA	11	3.6 (600 nm)	31
COF-BBT	2.0	Pt	AA	48.7	6.9 (420 nm)[f]	32
v-2D-COF-NO1	1.86	Pt	AA	1.97	-	33
COF-JLU35	1.85	Pt	AA	70.8	3.2 (500 nm)	34

Ni-COF-SCAU-1	-	Pt	AA	197.5	43.2 (420 nm)	35
BTT-BPy-PCOF	1.41	Pt	AA	15.8	3.7 (500 nm)[e]	36
PS-PEG5	-	Pt	AA	37.2	5.3 (365 nm)	37
F1 SC-NPs	-	Pt	AA	152.6	6.9 (600 nm)	38
PM6:PCBM	-	Pt	AA	73.7	8.7 (400 nm)	39
PTB7-Th/EH-IDTBR	-	Pt	AA	64.4	6.2 (700 nm)	1
CNP-f	2.51	Pt	AA	31.9	-	40
**TFP-BD nano-COF**	**2.20**	**Pt**	**AA**	**183**	**5.5 (420 nm)[f]**	**This work**
**TFP-BpyD nano-COF**	**2.24**	**Pt**	**AA**	**392**	**10 (420 nm)[f]**	**This work**

[a] Co-1: [Co(dmgH)$_2$pyCl]. [b]: 300 W Newport Xe light with a UV cutoff filter; [c]: solar simulator (AM1.5G); [d] PE: photonic efficiency. [e]AQE: apparent quantum efficiency. [f] EQE: external quantum efficiency. [g] QE: quantum efficiency; [h]: apparent quantum yield (AQY); TEOA: triethanolamine; TEA: triethylamine; AA: Ascorbic acid; SA: Sodium ascorbate; LA: Lactic acid. We note that absolute hydrogen evolution rates reported in different studies should be compared with caution because the details of the optical set up (light intensity, photolysis geometry, scale) are known to have a significant effect on rates. For the experiments performed here (*e.g.*, nano-COFs vs bulk COFs), all tests were made under the same photolysis conditions and they are hence directly comparable.

Supplementary Table 4. Quantum yields analysis of TFP-BpyD nano-COF.

TFP-BpyD nano-COF	Water	Quantum yields		Average quantum yields
0.1 mL	4.9 mL	Test 1	0.16%	0.17%
		Test 2	0.18%	
0.5 mL	4.5 mL	Test 1	0.12%	0.13%
		Test 2	0.13%	
1.0 mL	4.0 mL	Test 1	0.10%	0.10%
		Test 2	0.10%	

Note: Quantum yields of TFP-BpyD nano-COF at different concentrations followed a reverse concentration-dependent phenomenon as well. At lower concentration (0.1 mL), the quantum yield is 0.17 %. With the increase of concentration of TFP-BpyD nano-COF, the quantum yields decreased to 0.13 % (0.5 mL) and 0.10 % (1.0 mL).

2. References

1. Kosco, J. et al. Enhanced photocatalytic hydrogen evolution from organic semiconductor heterojunction nanoparticles. *Nat. Mater.* **19**, 559-565 (2020).
2. Zhao, W. et al. Using sound to synthesize covalent organic frameworks in water. *Nat. Synth.* **1**, 87-95 (2022).
3. Vyas, V. S. et al. A tunable azine covalent organic framework platform for visible light-induced hydrogen generation. *Nat. Commun.* **6**, 8508 (2015).
4. Ming, J. et al. Hot π-electron tunneling of metal-insulator-COF nanostructures for efficient hydrogen production. *Angew. Chem. Int. Ed.* **58**, 18290-18294 (2019).
5. Haase, F., Banerjee, T., Savasci, G., Ochsenfeld, C. & Lotsch, B. V. Structure-property-activity relationships in a pyridine containing azine-linked covalent organic framework for photocatalytic hydrogen evolution. *Faraday Discuss.* **201**, 247-264 (2017).
6. Banerjee, T. et al. Single-site photocatalytic H_2 evolution from covalent organic frameworks with molecular cobaloxime co-catalysts. *J. Am. Chem. Soc.* **139**, 16228-16234 (2017).
7. Li, L. et al. Thioether-functionalized 2D covalent organic framework featuring specific affinity to Au for photocatalytic hydrogen production from seawater. *ACS Sustainable Chem. Eng.* **7**, 18574-18581 (2019).
8. Biswal, B. P. et al. Sustained solar H_2 evolution from a thiazolo[5,4-d]thiazole-bridged covalent organic framework and nickel-thiolate cluster in water. *J. Am. Chem. Soc.* **141**, 11082-11092 (2019).
9. Wang, G. et al. A benzothiadiazole-based covalent organic framework for highly efficient visible-light driven hydrogen evolution. *Chem. Commun.* **56**, 12612-12615 (2020).
10. Chen, W. et al. Modulating benzothiadiazole-based covalent organic frameworks via halogenation for enhanced photocatalytic water splitting. *Angew. Chem. Int. Ed.* **59**, 16902-16909 (2020).
11. Bi, S. et al. Two-dimensional semiconducting covalent organic frameworks via condensation at arylmethyl carbon atoms. *Nat. Commun.* **10**, 2467 (2019).
12. Thote, J. et al. A covalent organic framework-cadmium sulfide hybrid as a

prototype photocatalyst for visible-light-driven hydrogen production. *Chem. Eur. J.* **20**, 15961-15965 (2014).

13. Dong, H. et al. Boosting visible-light hydrogen evolution of covalent-organic frameworks by introducing Ni-based noble metal-free co-catalyst. *Chem. Eng. J.* **379**, 122342 (2020).
14. Sheng, J. L. et al. Effect of different functional groups on photocatalytic hydrogen evolution in covalent-organic frameworks. *ChemCatChem*. **11**, 2313-2319 (2019).
15. Pachfule, P. et al. Diacetylene functionalized covalent organic framework (COF) for photocatalytic hydrogen generation. *J. Am. Chem. Soc.* **140**, 1423-1427 (2018).
16. Zhang, F. et al. Rational design of MOF/COF hybrid materials for photocatalytic H2 evolution in the presence of sacrificial electron donors. *Angew. Chem. Int. Ed.* **57**, 12106-12110 (2018).
17. Jin, E. et al. 2D sp^2 carbon-conjugated covalent organic frameworks for photocatalytic hydrogen production from water. *Chem*. **5**, 1632-1647 (2019).
18. Stegbauer, L., Schwinghammer, K. & Lotsch, B. V. A hydrazone-based covalent organic framework for photocatalytic hydrogen production. *Chem. Sci.* **5**, 2789-2793 (2014).
19. Wang, X. et al. Sulfone-containing covalent organic frameworks for photocatalytic hydrogen evolution from water. *Nat. Chem.* **10**, 1180-1189 (2018).
20. Wei, S. et al. Semiconducting 2D triazine-cored covalent organic frameworks with unsubstituted olefin linkages. *J. Am. Chem. Soc.* **141**, 14272-14279 (2019).
21. Gao, M. et al. Boosting visible-light-driven hydrogen evolution of covalent organic frameworks through compositing with MoS_2: a promising candidate for noble-metal-free photocatalysts. *J. Mater. Chem. A.* **7**, 20193-20200 (2019).
22. Ghosh, S. et al. Identification of prime factors to maximize the photocatalytic hydrogen evolution of covalent organic frameworks. *J. Am. Chem. Soc.* **142**, 9752-9762 (2020).
23. Stegbauer, L. et al. Tailor-made photoconductive pyrene-based covalent organic frameworks for visible-light driven hydrogen generation. *Adv. Energy Mater.* **8**, 1703278 (2018).
24. Wang, H. et al. Integrating suitable linkage of covalent organic frameworks into

covalently bridged inorganic/organic hybrids toward efficient photocatalysis. *J. Am. Chem. Soc.* **142**, 4862-4871 (2020).

25. Li, W. et al. Thiazolo[5,4-d]thiazole-based donor-acceptor covalent organic framework for sunlight-driven hydrogen evolution. *Angew. Chem. Int. Ed.* **60**, 1869-1874 (2021).
26. Zhao, Z. et al. Fabrication of robust covalent organic frameworks for enhanced visible-light-driven H2 evolution. *ACS Catal.* **11**, 2098-2107 (2021).
27. Zhang, W. et al. Reconstructed covalent organic frameworks. *Nature*. **604**, 72-79 (2022).
28. Li, Y. et al. In situ photodeposition of platinum clusters on a covalent organic framework for photocatalytic hydrogen production. *Nat. Commun.* **13**, 1355 (2022).
29. Liu, F. et al. Regulating excitonic effects in covalent organic frameworks to promote free charge carrier generation. *ACS Catal.* **12**, 9494-9502 (2022).
30. Ma, S. et al. Photocatalytic hydrogen production on a sp^2-carbon-linked covalent organic framework. *Angew Chem. Int. Ed.* **61**, e202208919 (2022).
31. He, T. et al. Integrated interfacial design of covalent organic framework photocatalysts to promote hydrogen evolution from water. *Nat. Commun.* **14**, 329 (2023).
32. Huang, W. et al. Highly crystalline and water-wettable benzobisthiazole-based covalent organic frameworks for enhanced photocatalytic hydrogen production. *Natl. Sci. Rev.* **10**, 171 (2023).
33. Li, S. et al. Direct construction of isomeric benzobisoxazole-vinylene-linked covalent organic frameworks with distinct photocatalytic properties. *J. Am. Chem. Soc.* **144**, 13953-13960 (2022).
34. Li, Z. et al. Three-component donor-π-acceptor covalent-organic frameworks for boosting photocatalytic hydrogen evolution. *J. Am. Chem. Soc.* **145**, 8364-8374 (2023).
35. Shen, R., Li, X., Qin, C., Zhang, P. & Li, X. Efficient photocatalytic hydrogen evolution by modulating excitonic effects in Ni-intercalated covalent organic frameworks. *Adv. Energy Mater.* **13**, 2203695 (2023).
36. Dai, L. et al. Enhancement of visible-light-driven hydrogen evolution activity of

2D π-conjugated bipyridine-based covalent organic frameworks via post-protonation. *Angew Chem. Int. Ed.* **62**, e202300224 (2023).

37. Yu, M., Zhang, W., Guo, Z., Wu, Y. & Zhu, W. Engineering nanoparticulate organic photocatalysts via a scalable flash nanoprecipitation process for efficient hydrogen production. *Angew. Chem. Int. Ed.* **60**, 15590-15597 (2021).
38. Zhu, Y. et al. Organic photovoltaic catalyst with extended exciton diffusion for high-performance solar hydrogen evolution. *J. Am. Chem. Soc.* **144**, 12747-12755 (2022).
39. Kosco, J. et al. Generation of long-lived charges in organic semiconductor heterojunction nanoparticles for efficient photocatalytic hydrogen evolution. *Nat. Energy*. **7**, 340-351 (2022).
40. Yang, H. et al. Packing-induced selectivity switching in molecular nanoparticle photocatalysts for hydrogen and hydrogen peroxide production. *Nat. Nanotechnol.* **18**, 307-315 (2023).
